# Supplementary material for: Rare variant contribution to human disease in 281,104 UK Biobank exomes
Source: Nature. 2021 Aug 10;597(7877):527–32. doi: 10.1038/s41586-021-03855-y (PMC8458098; doi:10.1038/s41586-021-03855-y)
Supplement: Supplementary file 1 — This file contains Supplementary Methods, Supplementary Figures 1-4, Supplementary Tables 3, 6, 9, 11, 13, 18, 21, 23, 25, and 27, detailed descriptions of Supplementary Datasets, and Supplementary References. [file 41586_2021_3855_MOESM1_ESM.docx]

**Rare variant contribution to human disease in 281,104 UK Biobank exomes**

***Supplemental Information***

Quanli Wang^1^*, Ryan S. Dhindsa^1^*, Keren Carss^2^*, Andrew R. Harper^2^, Abhishek Nag^2^, Ioanna Tachmazidou^2^, Dimitrios Vitsios^2^, Sri V.V. Deevi^2^, Alex Mackay^3^, Daniel Muthas^3^, Michael Hühn^3^, Sue Monkley^3^, Henric Olsson^3^, AstraZeneca Genomics Initiative^+^, Sebastian Wasilewski^2^, Katherine R. Smith^2^, Ruth March^4^, Adam Platt^5^, Carolina Haefliger^2^, Slavé Petrovski^2,6,#^

* These authors contributed equally

^#^ Corresponding author

^+^ A list of consortium authors and their affiliations appears at the end of the main text.

Affiliations

1. Centre for Genomics Research, Discovery Sciences, BioPharmaceuticals R&D, AstraZeneca, Waltham, USA
2. Centre for Genomics Research, Discovery Sciences, BioPharmaceuticals R&D, AstraZeneca, Cambridge, UK
3. Translational Science and Experimental Medicine, Research and Early Development, Respiratory and Immunology, BioPharmaceuticals R&D, AstraZeneca, Gothenburg, Sweden
4. Precision Medicine & Biosamples, Oncology R&D, AstraZeneca, Cambridge, UK
5. Translational Science and Experimental Medicine, Research and Early Development, Respiratory and Immunology, BioPharmaceuticals R&D, AstraZeneca, Cambridge, UK
6. Departments of Medicine and Neurology, University of Melbourne, Royal Melbourne Hospital, Melbourne, Victoria, Australia.
7. Translational Medicine, Research and Early Development, Oncology R&D, AstraZeneca, Waltham, Massachusetts, USA
8. Research and Early Development, Respiratory and Immunology, BioPharmaceuticals R&D, AstraZeneca, Cambridge, UK
9. Translational Genomics, Discovery Biology, Discovery Sciences, BioPharmaceuticals R&D, AstraZeneca, Gothenburg, Sweden
10. Biosciences CKD, Research and Early Development, Cardiovascular, Renal and Metabolism, BioPharmaceuticals R&D, AstraZeneca, Gothenburg, Sweden
11. Discovery Sciences, BioPharmaceuticals R&D, AstraZeneca, Gothenburg, Sweden
12. Research and Early Development, Cardiovascular, Renal and Metabolism, BioPharmaceuticals R&D, AstraZeneca, Gothenburg, Sweden
13. Oncology Discovery, Early Oncology, Oncology R&D, AstraZeneca, Cambridge, UK
14. Early Clinical Development, Research and Early Development, Cardiovascular, Renal and Metabolism, BioPharmaceuticals R&D, AstraZeneca, Gothenburg, Sweden
15. Translational Science & Experimental Medicine, Research and Early Development, Cardiovascular, Renal and Metabolism, BioPharmaceuticals R&D, AstraZeneca, Gothenburg, Sweden
16. Bioscience Asthma, Research and Early Development, Respiratory & Immunology, Biopharmaceuticals R&D, AstraZeneca, Cambridge, UK
17. Bioscience Asthma, Research and Early Development, Respiratory & Immunology, Biopharmaceuticals R&D, AstraZeneca, Gaithersburg US
18. Biopharmaceuticals R&D, AstraZeneca, Gaithersburg US

Contents

[Supplementary Methods: Parameters adopted from PHESANT package 4](#_Toc78239424)

[Supplementary Methods: Informativeness of individual genes 5](#_Toc78239425)

[Supplementary Methods: Identifying potential batch effect genes/variants 6](#_Toc78239426)

[Supplementary Methods: Evaluating suggestive signals in subsequent tranches 7](#_Toc78239427)

[Supplementary Methods: OMIM curation of Known, Expansion and Novel. 8](#_Toc78239428)

[Supplementary Methods: Publicly available databases. 9](#_Toc78239429)

[Supplementary Methods: Comparing 50K UKB gene-level results across multiple studies 10](#_Toc78239430)

[Supplementary Methods: Comparing ExWAS results using Fisher’s exact test to SAIGE SPA v0.43 and REGENIE v2.0.2 with covariates 12](#_Toc78239431)

[Supplementary Methods: ExWAS and Collapsing signal detection 15](#_Toc78239432)

[Supplementary Figure 1: Comparing SAIGE and Fisher’s exact test for Chapter IX variant-trait pairs with p-value < 1x10^-8^. 16](#_Toc78239433)

[Supplementary Figure 2: Comparing SAIGE, REGENIE, Fisher’s exact test for Chapter IX variant-trait pairs across MAF bins 17](#_Toc78239434)

[Supplementary Figure 3: Comparing MTR and non-MTR collapsing models 19](#_Toc78239435)

[Supplementary Figure 4: Somatic variants 20](#_Toc78239436)

[Supplementary Table 1 – Studied phenotypes 21](#_Toc78239437)

[Supplementary Table 2 – ExWAS top hits 21](#_Toc78239438)

[Supplementary Table 3 – ExWAS PTV signals achieving p-value <1x10^-8^ 22](#_Toc78239439)

[Supplementary Table 4 – ExWAS Missense Variants, GWAS annotated 25](#_Toc78239440)

[Supplementary Table 5 – ExWAS PTVs, GWAS annotated 25](#_Toc78239441)

[Supplementary Table 6 – Comparison between Fisher’s Exact Test, SAIGE and REGENIE 25](#_Toc78239442)

[Supplementary Table 7 – Lambda distributions from SAIGE, REGENIE, and Fisher’s exact test comparisons 26](#_Toc78239443)

[Supplementary Table 8 – Collapsing analysis top hits 26](#_Toc78239444)

[Supplementary Table 9 – Control-enriched collapsing analyses 27](#_Toc78239445)

[Supplementary Table 10 – OMIM status of significant binary collapsing analysis associations 28](#_Toc78239446)

[Supplementary Table 11 – Oncology aligned collapsing analyses summary 29](#_Toc78239447)

[Supplementary Table 12 – Oncology-related collapsing analysis associations 30](#_Toc78239448)

[Supplementary Table 13A: Significant gene-phenotype relationships from collapsing analysis also identified in ExWAS 31](#_Toc78239449)

[Supplementary Table 13B: ExWAS associations (MAF<0.1%) also identified in collapsing analysis 31](#_Toc78239450)

[Supplementary Table 14 – Non-intersecting PTV signals 32](#_Toc78239451)

[Supplementary Table 15 – Binary trait collapsing analyses in non-European populations 32](#_Toc78239452)

[Supplementary Table 16 - Pan-ancestry binary collapsing analysis 32](#_Toc78239453)

[Supplementary Table 17 - Pan-ancestry quantitative collapsing analysis 32](#_Toc78239454)

[Supplementary Table 18 – ExWAS n-of-1 permutation summary 33](#_Toc78239455)

[Supplementary Table 19 – ExWAS dominant model n-of-1 permutation results 33](#_Toc78239456)

[Supplementary Table 20 – Collapsing analysis null distribution 33](#_Toc78239457)

[Supplementary Table 21 – Synonymous collapsing output 34](#_Toc78239458)

[Supplementary Table 22 – Lambda distributions 34](#_Toc78239459)

[Supplementary Table 23 – Lambda ranges per collapsing model 34](#_Toc78239460)

[Supplementary Table 24 – Gene Informativeness 35](#_Toc78239461)

[Supplementary Table 25 - Gene-level cautions 36](#_Toc78239462)

[Supplementary Table 26 – Variant-level cautions 39](#_Toc78239463)

[Supplementary Table 27 - Variant-level exemptions 40](#_Toc78239464)

[Supplementary Table 28 – Cross-study comparison of 50K UKB exomes 43](#_Toc78239465)

[Supplementary Table 29 - Correlated Chapter IX phenotypes 43](#_Toc78239466)

[Supplementary References 44](#_Toc78239467)

# Supplementary Methods: Parameters adopted from PHESANT package

make_option(c("--catmultcutoff"), type="integer", default=10,
help="The cutoff for exclusion when creating dichotomous variables for CAT-multiple."),

make_option(c("--catordnacutoff"), type="integer", default=500,
help="The cutoff for exclusion for number of non-NAs in ordered categorical variables."),

make_option(c("--catunordnacutoff"), type="integer", default=500,
help="The cutoff for exclusion for number of non-NAs in unordered categorical variables."),

make_option(c("--contnacutoff"), type="integer", default=500,
help="The cutoff for exclusion for number of non-NAs in continuous variables."),

make_option(c("--binnacutoff"), type="integer", default=500,
help="The cutoff for exclusion for number of non-NAs in binary-variables."),

make_option(c("--bintruecutoff"), type="integer", default=30,
help="The cutoff for exclusion for numbers of members of a category in binary-variables."),

make_option(c("--mincategorysize"), type="integer", default=10,
help="The minimum number of samples in a category for categorical single, integer, and continuous variables."),

make_option(c("--maxunorderedcategories"), type="integer", default=1000,
help="The maximum number of categories in an unordered categorical variable"),

make_option(c("--propforcontinuous"), type="double", default=0.2,
help="The cutoff for proportion of samples with the same value for the variable to not be considered continuous.")

# Supplementary Methods: Informativeness of individual genes

Observing and equally importantly not observing signals among both gene- and variant-level association statistics are highly dependent on how informative the sequence data is for those regions of the exome.

We identified that the exome sequence data for UK Biobank participants who contributed to the analyses represented in this paper had on average 97.2% of the 34.1Mbp of the consensus coding sequence (CCDS release 22) covered with at least 10-fold coverage. To determine how informative individual genes were, we further broke down this assessment to the 18,762 genes represented in CCDS release 22. Overall, we found that 2,330 (12.4%) of genes had on average 100% of the protein-coding sites covered with at least 10x coverage among all UKB participants. A further 15,241 (81.2%) of genes had on average ≥99% of the protein-coding sites covered with at least 10x coverage among all UKB participants. An additional 856 (4.6%) of genes had on average ≥75% of the protein-coding sites covered with at least 10x coverage among the UKB participants. Leaving a remainder of 335 (1.8%) CCDS release 22 genes with < 75% of the protein-coding sites covered with at least 10x coverage among UKB participants. The individual gene-level coverage statistics are available in **Supplementary Table 24** and are a valuable resource for understanding the potential blind-spots in this UKB exome release.

# Supplementary Methods: Identifying potential batch effect genes/variants

Aside from the previously described preferential ascertainment for asthma and radiomics among the Regeneron-GSK initial 50K batch of exomes, the exome sequencing of the UK Biobank participants were randomly sampled tranches. This random sampling of participants is a critical component of the experimental design as it negates the potential impact of batch effects to the PheWAS test statistics. Nevertheless, in a conservative manner, we sought to identify potentially problematic genes and variants on basis of significant batch-assigned dummy variable enrichments and then mask their results from the PheWAS analyses.

To identify potential batch effects we created four dummy phenotypes to reflect each of the four individual exome sequencing tranches generated over time by Regeneron Pharmaceuticals and that make up the 300K exome release. We executed the variant-level ExWAS and the gene-level collapsing analyses under the identical parameterisations as the PheWAS.

Among the gene-based collapsing analyses, of the 18,762 studied genes we identified and excluded from PheWAS analyses 46 (0.25%) genes that achieved a gene-level collapsing analysis p<1x10^-7^ based on at least one of the twelve studied collapsing models (**Supplementary Table 25**). To determine whether any of these signals could be true biological enrichment due to the known asthma enrichment in the first 50K exomes batch we checked existing literature and none of these 46 genes have been previously associated with asthma.

Among the variant-based ExWAS analyses, of the 2,108,983 studied WES-based variants (as low as 0.001% MAF) we identified and excluded from ExWAS analyses 8,365 (0.40%) variants that achieved a variant-level ExWAS statistic p<1x10^-6^ based on either allelic, dominant or recessive genetic models (**Supplementary Table 26**). Despite not satisfying the p>1x10^-6^ criteria, thirteen variants were retained in the ExWAS analyses on the basis of being previously reported in ClinVar database (accessed 2^nd^ July 2020)^1^ (**Supplementary Table 27**).

# Supplementary Methods: Evaluating suggestive signals in subsequent tranches

Exome sequence data was obtained in discrete batches, with the cumulative total number of available exomes increasing from 50k to 300k. Additionally, health outcomes data was updated in July 2020 and facilitated a second analysis of the 300k tranche. A *P* threshold of *<* 2x10^-9^ was considered significant, suggestive was defined as 1x10^-7^ < *P* < 2x10^-9^ and non-significant reported as *P* > 1x10^-7^. All model-specific gene-phenotype relationships were harmonised across four tranches (50k, 150k, 300k_v1 and 300k_v2). The outcome of gene-phenotype-model relationships that appeared suggestive (i.e. 1x10^-7^ < *P* < 2x10^-9^) in the *i^th^* tranche were evaluated in the *i+1* tranche.

We observed that many statistically suggestive (i.e., within 2 x 10^-9^ < P < 1 x 10^-7^ range) gene-phenotype associations represent true positive associations. Looking at the patterns across three UKB exome sequencing tranches we found that the proportion of suggestive associations for binary traits that achieve statistical significance in subsequent tranches is consistently high. For instance, two-thirds of suggestive associations reported in the 150K UKB exomes tranche achieved statistical significance in the 300Kv1 tranche, which includes phenotypic data released up to April 2017 (**Extended Data Fig. 5c**).

# Supplementary Methods: OMIM curation of Known, Expansion and Novel.

All binary trait gene-phenotype relationships demonstrating *P* <2x10^-9^ in the 300K_v2 PheWAS, from any of the 11 non-synonymous collapsing analysis models, were identified (n=2,856). Gene-phenotype relationships were collapsed by gene (n=82). Genes located within the major histocompatibility complex region (defined here as Chr6:25Mbp-35Mbp) were excluded. The novelty of each gene was then evaluated by comparing the associated phenotypes from the 300k_v2 PheWAS with prior literature reports curated by the Online Mendelian Inheritance in Man catalogue (accessed December 5^th^ 2018) (OMIM).^2^ The status of each gene was determined as either: known (reported gene-phenotype relationship previously documented in OMIM); novel (no prior evidence for the reported gene having a disease relationship in OMIM); or, expanded (the gene has previously been reported with a phenotype in OMIM, but the gene-phenotype relationship reported in the 300k_v2 PheWAS extends beyond this known association) (**Supplementary Table 10**).

# Supplementary Methods: Publicly available databases.

We queried a number of publicly available databases to determine whether genes that achieved study-wide significant missense and/or protein-truncating variants identified in the ExWAS mapped to genes previously reported in relation to a comparable phenotype. This included resources:
(i) OMIM (<https://omim.org>)
(ii) ClinVar (<https://www.ncbi.nlm.nih.gov/clinvar/>(iii) FinnGen release 5 ([http://r5.finngen.fi](http://r4.finngen.fi))
(iv) EBI GWAS catalogue (<https://www.ebi.ac.uk/gwas/>)

In the case of ClinVar, we focussed on the variants annotated as likely pathogenic as captured by ClinVar’s ‘ClinSigSimple’ filter. In the FinnGen data, we considered missense variants and PTVs that were associated with the same/comparable phenotype and achieved a p-value < 1x10^-4^.

# Supplementary Methods: Comparing 50K UKB gene-level results across multiple studies

The first tranche of 50,000 UKB exomes was initially analysed by Van Hout et al under a collaborative effort between Regeneron Genetics Centre and GlaxoSmithKline,^3^ and later on by Cirulli et al within the auspices of Helix (https://ukb.research.helix.com/).^4^ Both groups performed gene-level collapsing analyses to explore the aggregate effect of rare variants on thousands of binary and quantitative traits found in the UKB repository. Their and our efforts differ in terms of the choice of variants collapsed, filters such as MAF, choice of phenotypes studied and statistical models. Here we endeavour to list the differences in methodology and subsequent results from the three efforts.

Van Hout et al^3^ used 49,960 individuals of European ancestry and focused on autosomal genes with > 3 predicted loss of function (pLoF) variants with MAF ≤ 1%. They run an additive collapsing model such that any individual that is heterozygous for at least one qualifying LoF in that gene region is considered heterozygous, and individuals that carry two copies of the same LoF are considered homozygous. Quantitative measures with ≥ 5 individuals were rank-based inverse normal transformed and analysed using BOLT-LMM v2.^5^ Prior to normalization, traits were first transformed as appropriate (log10, square) and adjusted for a standard set of covariates including age, sex, study site, first four principal components of ancestry, and in some cases BMI and/or smoking status. Data-points greater than five median absolute deviations from the median were excluded as outliers prior to normalization. Binary traits were based on ICD-10 diagnosis (primary diagnosis or ≥ 2 secondary diagnoses in in-patient Health Episode Statistics records), self-reported illness from verbal interview and physician-diagnosed illness. Binary outcomes with ≥ 50 cases were assessed with covariate adjustment for age, sex and first four principle components of ancestry using a generalized mixed model implemented in SAIGE.^6^

Cirulli et al^4^ used a dominant model and collapsed: 1) LoF variants only, and 2) coding variants only and not Polyphen or SIFT benign. The qualifying variants had MAF < 0.1% in the European ancestry UK Biobank unrelated set, as well as in any gnomAD population. Phenotypes were processed using the Neale lab modified version of PHESANT^7^. Statistical analysis was performed by BOLT-LMM,^5^ adjusting for age and sex. Genes needed to have at least five carriers of qualifying variants for quantitative traits and at least ten carriers of qualifying variants to be expected in the smaller sample group for binary traits. The traits that failed to run with BOLT-LMM (trait heritability fell below the required algorithm threshold) were analysed by linear regression using plink (quantitative traits adjusted for age, sex and the first 10 European-specific principle components) and Fisher’s exact test (binary traits) in the subset of unrelated European ancestry individuals. The Fisher’s exact test was also used to identify associations when <10 cases were expected to be carrying qualifying variants based on the overall prevalence. Although Cirulli et al^4^ analysed all ancestries on top of the European ancestry-only subset, here we focused on the results using European ancestry only.

Focusing on the initial 50K exomes compared to these two ground-breaking UKB PheWAS studies^3,4^ on the initial 50K exomes, in our study we found 62 statistically significant (*P* < 3.4x10^-10^ adopted by^4^) gene-trait pairs (5 for binary and 57 for quantitative outcomes), while Van Hout et al^3^ identified 11 gene-trait pairs (2 binary and 9 quantitative) and Cirulli et al^4^ reported 51 gene-trait pairs (7 binary and 44 quantitative phenotypes) (Tables 5 & 6 from^3^; Supplementary Data 2 from^4^; **Supplementary Table 28**). Across the 81 unique statistically significant gene-trait associations found among phenotypes analysed by the three studies, 24 were exclusively identified in this study, 14 exclusively by Cirulli et al^4^ and 5 exclusively by Van Hout et al^3^ (**Extended Data Figure 5b; Supplementary Table 28**).

# Supplementary Methods: Comparing ExWAS results using Fisher’s exact test to SAIGE SPA v0.43 and REGENIE v2.0.2 with covariates

We took all 324 binary “Union” traits from Chapter IX Diseases of the circulatory system and all variants on chromosomes 1-22, to compare ExWAS results from the Fisher’s exact test with results from SAIGE [version 0.43].^6^ For the purposes of this comparison, we took the same samples that were used with Fisher’s exact test and identified their genotype data. From these data, we selected high quality markers as input to SAIGE in order to create a kinship matrix. The criteria for selecting high quality markers were those used by Bycroft et al,^8^ i.e., autosomal directly-typed variants on both UKBL and UKBB arrays that passed variant QC in all batches, MAF >= 1%, HWE p-value ≥ 1x10^-6^, missingness <= 2% across arrays, not multiallelic, not indels, not C/G or A/T, not in regions of long-range LD or commonly inverted regions (23 regions from Table S12 in Bycroft et al), no variants with different allele frequencies between UKBL and UKBB, no variants with different allele frequencies between UKBB and HRC, and finally pruned for LD *r^2^*<0.1. There were 478,926 variants that went into LD pruning, resulting in 145,962 pruned markers. As covariates in SAIGE we used age, sex, sequencing batch and the 10 PCs as provided by Bycroft et al. Following advice from the SAIGE website, we restricted the results to those variants with minor allele count (MAC) ≥ 3. We also subsequently repeated the SAIGE runs for chromosome 1 only without adjusting for PCs. For the comparison, we used the summary statistics from SAIGE that implement the saddle-point approximation to account for case-control imbalance, which ranges between 1.12 to 8957 with a median of 700 for traits in Chapter IX. Our observations from this effort are:

1. Across the 324 traits, there are 5,541 trait-genotype pairs with SAIGE p-value is 0. In all cases, MAC within cases or controls is zero. We conclude that the saddle-point approximation implemented in SAIGE is unstable when either MAC within cases or MAC within controls is < 3.
2. There are 401 variant-trait pairs with p-value < 1x10^-8^ from SAIGE with PCs (351 associations at common variants (MAF≥0.05), 35 associations at low frequency variants (0.01≤MAF<0.05) and 15 associations at rare variants (MAF<0.01)). 41/401 associations were above this p-value threshold using Fisher’s exact test (35 associations at common variants, 1 at low frequency and 5 rare). Similarly, there are 439 variant-trait pairs with p-value < 1x10^-8^ from Fisher’s exact test (386 associations at common variants, 34 at low frequency variants and 19 at rare variants). 79/439 of these associations were above this p-value threshold using SAIGE (70 associations at common variants and 9 at rare variants) (**Supplementary Table 6**). Then there are 360 variant-trait pairs with p-value < 1x10^-8^ for both SAIGE and Fisher’s exact test (316 associations at common variants, 34 at low frequency variants and 10 at rare variants). The Pearson’s correlation coefficient (*r*) between the (-10*log_10_[p-values]) Phred values from SAIGE (with covariates) and the Fisher’s exact test for those 360 variants was 0.9951161 with 95% confidence interval 0.9939935-0.9960293. Moreover, there are 480 trait-genotype pairs with either SAIGE or Fisher’s exact test have p-value < 1x10^-8^. The Pearson’s correlation coefficient between the Phred values from SAIGE and the Fisher’s exact test for those 480 variants was 0.9901426 with 95% confidence interval 0.9882161-0.9917554 (**Supplementary Figure 1**).
3. There are 654,927,125 variant-trait pairs where both FET and SAIGE return a p-value (after excluding the pairs with SAIGE p-value=0). Pearson’s correlation coefficient on these (-10*log_10_[p-values]) Phred values within trait ranges between 83-98% (**Supplementary Table 7**), and a Pearson’s r of 95% across all ~655M variant-trait pairs. An explanation for the highly correlated results include: 1) we have been very careful in selecting predominantly unrelated samples with genetic ancestry harmonisation as part of the study design, and 2) we have down-sampled controls in each case-control configuration where there was a significant difference (Fisher’s exact p<0.05) to balance male and female ratios in case and control groups. A subset of 2,105,332 variant-trait pairs have p-values < 0.01. Overall, we identified that the SAIGE p-values in this 1x10^-8^ < p-values < 0.01 range achieve lower p-values than the Fisher’s exact test (Wilcoxon test: W = 7.87e+11, p-value < 2.2x10^-16^). When focusing across different MAF ranges: common (MAF≥0.05), low frequency (0.01≤MAF<0.05), rare (0.001≤MAF<0.01) and very rare (MAF<0.001) variants (**Supplementary Figure 2a-2d**), SAIGE reports lower p-values than the Fisher’s exact test statistic with decreasing MAF. We therefore reach the conclusion that in this setting the Fisher’s exact p-values are more conservative than SAIGE.
4. Pearson’s correlation coefficient between (-10*log_10_[p-values]) Phred values from SAIGE with 10 PCs from Bycroft et al. versus SAIGE with no PCs ranges between 0.8425 to 0.9998 with a median of 0.99. This result is based on chromosome 1 only and reflects the fact that: 1) we have carefully selected samples of European genetic ancestry, and 2) adjusting for PCs might not be necessary once we have adjusted for the kinship matrix in SAIGE. Adding 10 PCs as covariates in this situation is a conservative approach, which increases SAIGE running times by 1.5-fold.
5. Recognizing that there also exists correlation between phenotypes, we calculated pairwise correlation between all pairs of Chapter IX phenotypes included in this analysis. Among 53,301 phenotype pair-wise comparisons, 140 pairs achieved a Pearson’s *r*^2^ > 0.2 and only 71 achieved an *r*^2^ > 0.4, suggesting that most of the phenotypes adopted for this comparison are not strongly correlated. Phenotype pairs with an r^2^ > 0.2 as **Supplementary Table 29**.

In summary, we: 1) find a Pearson’s *r* of 0.99 for variant-phenotype associations in the statistical range of greatest interest p<1x10^-8^, 2) find that for low frequency variants (MAF≤0.01) the Fisher’s exact test generates more conservative p-values than the SAIGE SPA test with covariates, 3) find that the exact test is robust even when MAC approaches zero in cases/controls and 4) find that SAIGE has a ~29.5-fold increase in CPU run time per association test, which correlates to lower sustainability and increased costs compared to the Fisher’s exact tests **(Supplementary Table 6)**.

We subsequently took the same 324 binary “Union” traits from Chapter IX Diseases of the circulatory system and all variants on chromosomes 1-22, to compare ExWAS results from the Fisher’s exact test with results from REGENIE [version 2.0.2].^9^ We adopted the same high quality markers described for SAIGE exercise as input to REGENIE in order to create a kinship matrix. Following advice from the REGENIE website, we adopted the --firth --approx options to run the Firth likelihood ratio test (switching to the approximate Firth ratio test for p-values < 0.05). We ran REGENIE 2.0.2 step 1 with all 324 traits in one analysis and ran REGENIE 2.0.2 step 2 with 5 traits per analysis to balance between the CPU run time and elapsed run time. We adjusted REGENIE 2.0.2 with the same covariates as SAIGE, i.e., age, sex, sequencing batch and 10 PCs as provided by Bycroft et al. Following additional advice from the REGENIE website, we restricted comparisons to only variants with MAC ≥ 5. Our observations from this effort are summarised in **Supplementary** **Table 6**. In summary, we: 1) find a Pearson’s *r* of 0.99 for variant-phenotype associations in the statistical range of greatest interest p<1x10^-8^, 2) find that for low frequency variants (MAF≤0.01) the Fisher’s exact test generates more conservative p-values than the REGENIE v2.0.2 test with covariates (**Supplementary** **Figure 2**), and 3) find that REGENIE 2.0.2 has a ~11.2-fold increase in CPU run time per association test in comparison to the Fisher’s Exact tests.

# Supplementary Methods: ExWAS and Collapsing signal detection

Focusing on the PTV collapsing models, we also examined the proportion of the statistically significant gene-trait associations identified using a more generous MAF filter of 5% (“ptv5pcnt” model) that were not detected by both the stricter MAF threshold of 0.1% (“ptv” model) and full set of ExWAS results. We observe that 12.0% (49/410) and 12.1% (83/670) of gene-trait associations captured by the “ptv5pcnt” model were not captured by either the “ptv” model or ExWAS for quantitative and binary traits, respectively (**Supplementary Table 14**). Thus, while variant-level analyses are best suited for identification of common variant associations, collapsing analysis based on slightly relaxed MAF thresholds—especially for PTVs for which the functional effect is better understood—can yield additional novel associations. Among the distinct gene-phenotype relationships identified through the collapsing analysis for binary traits (barring the operation codes traits), 17% (125/724) were also detected using ExWAS, indicating that the remaining 83% were undetectable by traditional variant-level association tests. For quantitative traits, 58% (446/767) of the collapsing analysis associations were detectable using ExWAS (**Supplementary Table 13A**). This highlights that, particularly in the case-control scenario, a gene-based collapsing framework can identify associations that are currently undetectable by single variant-based approaches in an identical test setting. Correspondingly, a majority of the significant collapsing analysis associations that were not detected in ExWAS—83% (499/599) for binary traits and 68% (219/321) for quantitative traits—were captured by the rare (MAF<0.1%) missense and PTV collapsing models. When considering the rare PTV (MAF<0.1%) associations from ExWAS, we found that most of the gene-phenotype relationships were also captured in the PTV collapsing analysis (91% [83/91] for quantitative and 83% [30/36] for binary traits). The corresponding rates were lower for missense variants (45% [122/270] for quantitative and 23% [42/182] for binary traits) (**Supplementary Table 13B**).

# Supplementary Figure 1: Comparing SAIGE and Fisher’s exact test for Chapter IX variant-trait pairs with p-value < 1x10^-8^.


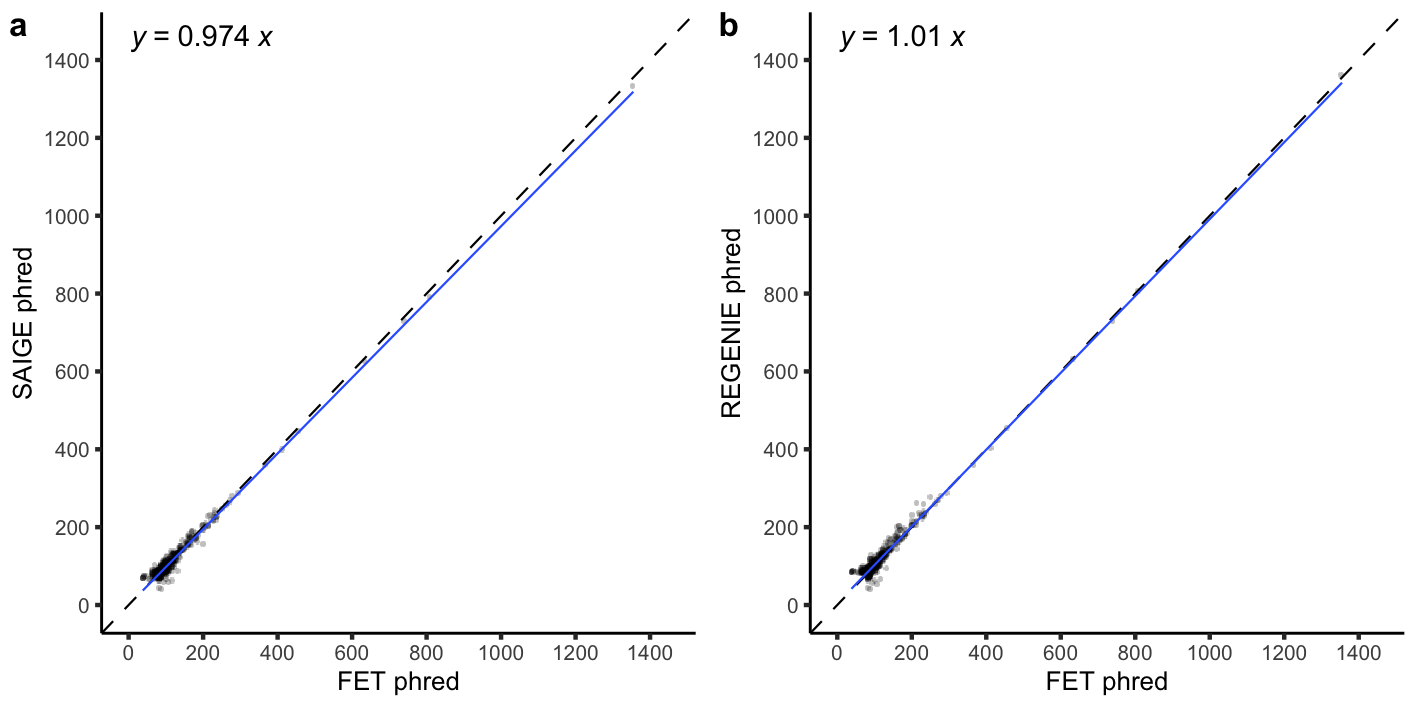


**(a)** Scatter plot depicting SAIGE and the Fisher’s exact test PHRED scores (-10*log_10_(p-value)) for Chapter IX autosomal variant-trait pairs achieving a p-value < 1x10^-8^ in either test. Pearson’s correlation coefficient is 0.9916 with 95% confidence interval 0.9899-0.9930. **(b)** Scatter plot depicting SAIGE and the Fisher’s exact test PHRED scores (-10*log_10_(p-value)) for Chapter IX autosomal variant-trait pairs achieving a p-value < 1x10^-8^ in either test. Pearson’s correlation coefficient is 0.9884 with 95% confidence interval 0.9862-0.9902. We included age, sex, sequencing batch and the 10 PCs as provided by Bycroft et al. as covariates for both SAIGE and REGENIE. FET = Fisher’s exact test. Included p-values are not corrected for multiple testing; the study-wide significance threshold is p ≤ 2 ×10^-9^. FET p-values were generated via a two-tailed Fisher’s exact test. SAIGE and REGENIE rely on regression frameworks to generate p-values.

Supplementary Figure 2: Comparing SAIGE, REGENIE, Fisher’s exact test for Chapter IX variant-trait pairs across MAF bins


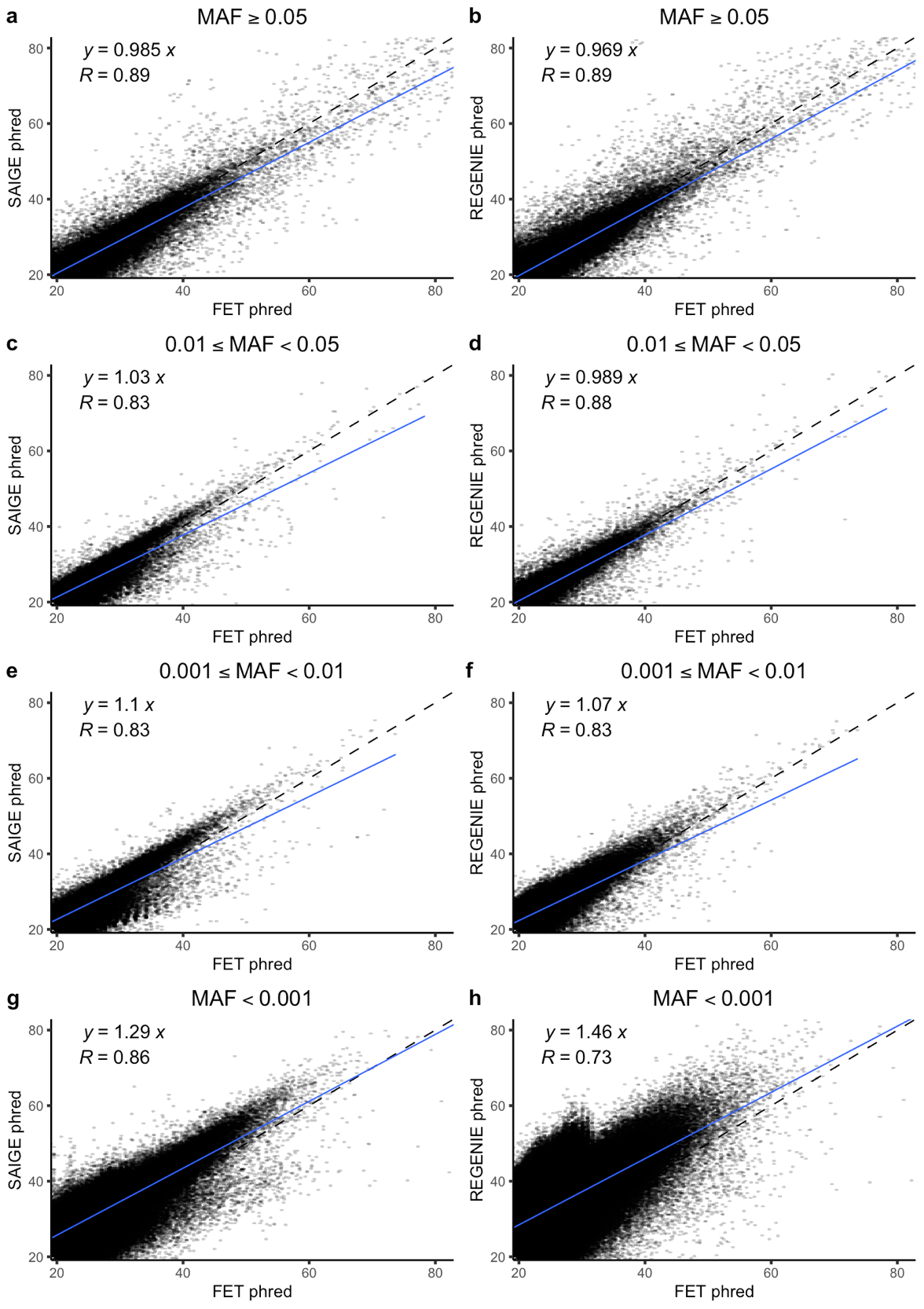


Scatter plots depicting SAIGE, REGENIE, and the Fisher’s exact test Phred scores (-10*log_10_(p-value) for Chapter IX autosomal variant-trait pairs. We included age, sex, sequencing batch and the 10 PCs as provided by Bycroft et al. as covariates for both SAIGE and REGENIE. In each plot, variants are limited to those achieving a 1x10^-8^ < p-value < 0.01 in either test. Variants are depicted separately for common **(a, b)**, low frequency **(c, d)**, rare **(e, f)** and very rare **(g, h)** variants. Linear regression lines are indicated in blue and Pearson’s *r* is annotated in each panel. Axes are capped at 20 and 80. Included p-values are not corrected for multiple testing; the study-wide significance threshold is p≤2 ×10^-9^. FET = Fisher’s exact test. FET p-values were generated via a two-tailed test. SAIGE and REGENIE rely on regression frameworks to generate p-values.

Supplementary Figure 3: Comparing MTR and non-MTR collapsing models

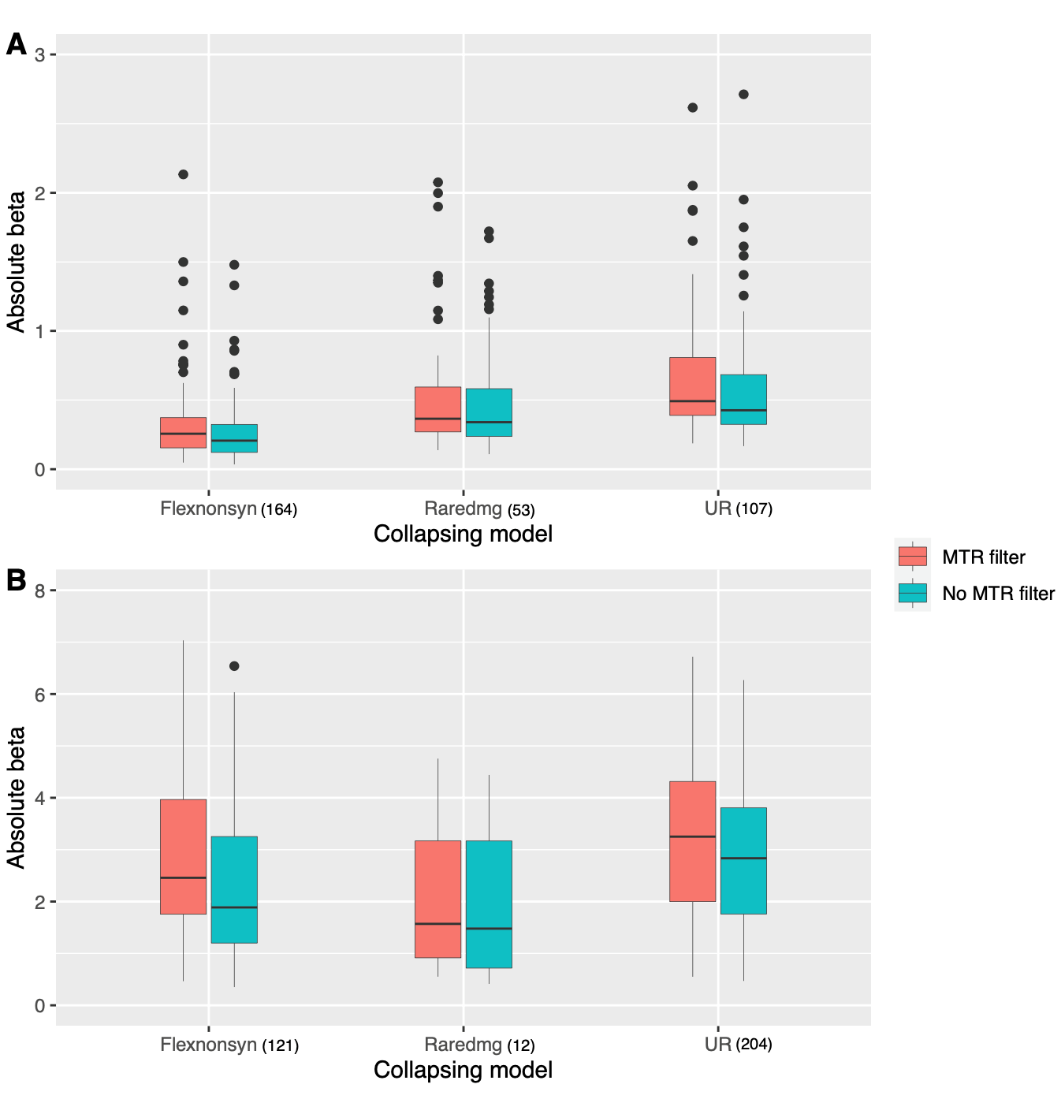


**Supplementary Figure 3. Comparing the absolute effect sizes between the ‘mtr‘ and the ‘non-mtr‘ versions of the collapsing models.** Using the set of gene-phenotype relationships common to each pair. **(A)** Quantitative traits **(B)** Binary traits.
The numbers in the parenthesis adjacent to the model names refer to the gene-phenotype relationships that were common to the MTR-informed and MTR-uninformed versions of the corresponding model, which are as follows: (i) Quantitative traits: flexnonsyn (N=164), raredmg (N=53) and UR(107) (ii) Binary traits: flexnonsyn (N=121), raredmg (N=12) and UR (204). Boxplots show median (centre line) and interquartile range (IQR) (box limits); the length of the whiskers corresponds to 1.5 × IQR; the outlier values indicate those >1.5 times and <3 times the IQR beyond either end of the box.

# Supplementary Figure 4: Somatic variants

Distribution of percent of reads mapping to the alternate allele for haematological malignancies. *BRCA1* and *BRCA2* are included as controls. Except for *DDX41*, these distributions suggest that our pipeline detected somatic variants in these genes.

# Supplementary Table 1 – Studied phenotypes

Provided as separate additional file

Details of all phenotypes studied in this dataset. Binary: 17,361 binary phenotypes used for both gene-level collapsing analysis and variant-level ExWAS. Path, Root, and Field refer to UKB data structure as described in Showcase here <https://biobank.ndph.ox.ac.uk/showcase/browse.cgi>. For phenotypes where Path and Field is “Union”, please see “Union Mapping” tab for details. 300Kv2 refers to the UKB phenotypes up to and including those released in July 2020. Phenotypes where controls were restricted to individuals without a diagnosis in the chapter (“Smart Controls”) and phenotypes where controls were down-sampled for case-control sex-matching (“Sex Matching”) are also flagged. Quantitative: 1,419 quantitative traits used for both gene-level collapsing analysis and variant-level ExWAS. Union Mapping: The binary phenotype fields from UKB that were combined to create union phenotypes. 41270_Refresh: For each phenotype in UKB field 41270 (Diagnoses - ICD10), number of diagnoses before and after the July 2020 release, showing the % increase in diagnoses gained by this update.

# Supplementary Table 2 – ExWAS top hits

Provided as separate additional file

Most significant genotype-phenotype associations from variant-level exome-wide association study. ExWAS_binary: binary associations (all variant consequences) excluding MHC region (chr6:25Mbp-35Mbp) with p<5x10^-8^. ExWAS_binary_MHC_region: binary associations in the MHC region (chr6:25Mbp-35Mbp) with p<5 x10^-8^. ExWAS_binary_PTV: The subset of ExWAS_binary that are protein-truncating variants with p≤2x10 ^-9^. ExWAS_binary_missense: The subset of ExWAS_binary that are missense variants with p ≤2x10 ^-9^. ExWAS_quantitative: quantitative associations (all variant consequences) excluding MHC region (chr6:25Mbp-35Mbp) with p≤2x10 ^-9^. P-values were generated via two-tailed Fisher’s exact test (binary traits) or linear regression (quantitative traits). Included p-values are not corrected for multiple testing; the study-wide significance threshold is p≤2 ×10^-9^.

# Supplementary Table 3 – ExWAS PTV signals achieving p-value <1x10^-8^

| **Gene** | **Variant** | **Most Damaging** | **model** | **Phenotype** | **p-value** | **OR** | **OR LCI** | **OR UCI** | **Case MAF** | **Ctrl MAF** | **ClinVar** | **EBI GWAS** | **OMIM** |
| --- | --- | --- | --- | --- | --- | --- | --- | --- | --- | --- | --- | --- | --- |
| *HBB* | 11-5226774-G-A | stop gained | dom | Union# D56 Thalassaemia | 2.1E-26 | 2341.9 | 948.5 | 5782.3 | 0.0437 | 2.0E-05 | 1 | Yes | Delta-beta thalassemia |
| *FLG* | 1-152312600-CACTG-C | frameshift | rec | 20002# eczema\|dermatitis | 4.8E-22 | 9.9 | 6.8 | 14.3 | 0.0454 | 0.0235 | 1 | NA | Ichthyosis vulgaris |
| *FES* | 15-90885291-CT-C | frameshift | allele | Union#I10#I10 Essential (primary) hypertension | 4.7E-22 | 1.06 | 1.05 | 1.08 | 0.334 | 0.321 | NA | Yes | NA |
| *COL4A4* | 2-227052367-G-C | stop gained | dom | Union# R31 Unspecified haematuria | 2.2E-21 | 5.2 | 3.9 | 7.0 | 0.0024 | 0.0005 | 1 | NA | Hematuria; familial benign |
| *TSHR* | 14-81143695-G-A | stop gained | dom | Source of report of E03 (other hypothyroidism) | 6.0E-15 | 4.4 | 3.1 | 6.1 | 0.0014 | 0.0003 | 1 | NA | Hyperthyroidism; familial gestational |
| *CLECL1* | 12-9733111-A-ATAAGT | frameshift | rec | 20002# hypothyroidism\|myxoedema | 1.4E-14 | 0.84 | 0.81 | 0.88 | 0.4706 | 0.4978 | NA | NA | NA |
| *IL33* | 9-6255967-G-C | splice acceptor | dom | 20002# 1111# asthma | 7.4E-14 | 0.57 | 0.48 | 0.67 | 0.0026 | 0.0046 | NA | Yes | NA |
| *^UMOD* | 16-20349020-CA-C | frameshift | dom | 41202# N18.5 Chronic kidney disease\| stage 5 | 2.7E-13 | 2357.9 | 274.7 | 20236.8 | 0.0073 | 3.1E-06 | NA | NA | Glomerulocystic kidney disease with hyperuricemia and isosthenuria |
| *CHEK2* | 22-28695868-AG-A | frameshift | allele | 20001# breast cancer | 2.7E-13 | 3.1 | 2.4 | 4.0 | 0.0053 | 0.0017 | 1 | NA | {Breast and colorectal cancer; susceptibility to} |
| *MROH2A* | 2-233823665-G-A | splice donor | dom | E80 (disorders of porphyrin and bilirubin metabolism) | 2.8E-12 | 1.7 | 1.4 | 1.9 | 0.2251 | 0.158 | NA | NA | NA |
| *NEK9* | 14-75101655-A-G | splice donor | dom | 41272# Y80.1 Inhalation anaesthetic using muscle relaxant | 1.2E-10 | 5.4 | 3.6 | 8.1 | 0.0071 | 0.0013 | NA | NA | Lethal congenital contracture syndrome |
| *PALB2* | 16-23621362-C-T | stop gained | dom | 41200# B27 Total excision of breast | 2.8E-10 | 8.3 | 4.9 | 13.8 | 0.0028 | 0.0003 | 1 | NA | {Breast cancer; susceptibility to} |
| *ACSM1* | 16-20627254-A-T | splice donor | dom | Union# N60 Benign mammary dysplasia | 3.0E-10 | 0.86 | 0.82 | 0.90 | 0.2997 | 0.3232 | NA | NA | NA |
| *MUC1* | 1-155192276-C-T | splice acceptor | rec | Union# K31.7 Polyp of stomach and duodenum | 3.2E-10 | 0.81 | 0.75 | 0.86 | 0.4294 | 0.4632 | NA | Yes | Medullary cystic kidney disease |
| *NOD2* | 16-50729867-G-GC | frameshift | rec | 20002# crohns disease | 3.6E-10 | 33.5 | 16.0 | 70.1 | 0.0561 | 0.018 | 0 | NA | {Inflammatory bowel disease 1; Crohn disease} |
| *LPL* | 8-19962213-C-G | stop gained | dom | E78 (disorders of lipoprotein metabolism and other lipidaemias) | 3.8E-10 | 0.92 | 0.90 | 0.95 | 0.092 | 0.098 | 0 | Yes | Combined hyperlipidemia; familial; 144250 (3); |
| *RP1* | 8-54625911-C-T | stop gained | dom | Union# H35.5 Hereditary retinal dystrophy | 8.8E-10 | 2893.2 | 576.5 | 14520.6 | 0.0156 | 5.6E-06 | 1 | NA | Retinitis pigmentosa |
| *ARMS2* | 10-122454839-C-T | stop gained | dom | Union# G56.0 Carpal tunnel syndrome | 1.2E-09 | 0.85 | 0.81 | 0.90 | 0.1368 | 0.1532 | 0 | NA | {Macular degeneration; age-related; 8} |
| *ABO* | 9-133257521-T-TC | splice acceptor | dom | 41202# I80.1 Phlebitis & thrombophlebitis of femoral vein | 1.3E-09 | 2.4 | 1.8 | 3.2 | 0.4716 | 0.3335 | NA | NA | [Blood group; ABO system] |
| *BRCA2* | 13-32379464-AC-A | frameshift | dom | 41202# Z40.0 Prophylactic surgery for risk-factors related to malignant neoplasms | 1.4E-09 | 641.6 | 117.2 | 3511.6 | 0.0042 | 6.6E-06 | 1 | NA | Multiple Cancers |
| *BRCA1* | 17-43092848-GTT-G | frameshift | dom | 41202# Z40.0 Prophylactic surgery for risk-factors related to malignant neoplasms | 1.4E-09 | 641.6 | 117.2 | 3511.6 | 0.0042 | 6.6E-06 | 1 | NA | Multiple Cancers |
| *PSG4* | 19-43203885-C-T | splice donor | allele | 41272# O12.1 Superficial temporal artery | 1.7E-09 | 370.4 | 111.2 | 1233.7 | 0.0075 | 2.0E-05 | NA | NA | NA |
| *RAD51B* | 14-67864961-C-CTT | splice acceptor | allele | Union# J18.9 Pneumonia\| unspecified | 3E-09 | 211.2 | 25.4 | 1754.5 | 0.0005 | 2.5E-06 | NA | NA | NA |
| *AIFM1* | X-130149545-A-G | stop lost | dom | 41202# M72.0 Palmar fascial fibromatosis [Dupuytren] | 3.1E-09 | 0.8 | 0.75 | 0.86 | 0.3553 | 0.4009 | 0 | NA | Combined oxidative phosphorylation deficiency \| Cowchock syndrome |
| *IFT140* | 16-1557934-C-A | splice donor | dom | Source of report of Q61 (cystic kidney disease) | 3.2E-09 | 18.7 | 9.5 | 36.5 | 0.0092 | 0.0005 | 1 | NA | Retinitis pigmentosa |
| *ATXN2L* | 16-28835545-G-A | splice acceptor | dom | Union# E66 Obesity | 3.3E-09 | 1.1 | 1.1 | 1.1 | 0.4111 | 0.3981 | NA | NA | NA |
| *NACA2* | 17-61590592-G-A | stop gained | allele | 41202# M16 Coxarthrosis [hip arthrosis] | 5.3E-09 | 1.1 | 1.1 | 1.2 | 0.163 | 0.1483 | NA | NA | NA |
| *TG* | 8-132882609-C-T | stop gained | allele | Source of report of E03 (other hypothyroidism) | 5.6E-09 | 2.6 | 1.9 | 3.5 | 0.0016 | 0.0006 | 1 | Yes | Thyroid dyshormonogenesis |
| *GCSAML* | 1-247556467-G-A | splice donor | allele | Union# L50 Urticaria | 6.7E-09 | 1.2 | 1.1 | 1.3 | 0.0822 | 0.0683 | NA | Yes | NA |
| *MYOC* | 1-171636338-G-A | stop gained | dom | 41200# C60 Filtering operations on iris | 7.3E-09 | 8.0 | 4.7 | 13.7 | 0.0103 | 0.0013 | 1 | Yes | Glaucoma 1A; primary open angle |
| *OR4X2* | 11-48245184-C-G | stop gained | allele | 20002# hypertension | 9.5E-09 | 0.95 | 0.93 | 0.97 | 0.1359 | 0.1423 | NA | NA | NA |

ExWAS protein-truncating variant signals that achieved a p-value < 1x10^-8^ in at least one of the three studied genetic models for binary traits (allelic, dominant or recessive). Not including variants within the MHC region, defined here as chr6:25Mbp–35Mbp. For table brevity, only the most significant association is presented for each pair of gene and closely related phenotypes. Full phenotypic description is provided here to allow the reader to connect these data to those in **Supplementary Table 1**. One indel of length >10bp and six indel variants with HWE Exact p-values <1x10^-7^ for both cases and controls were omitted. Shaded lines = control MAF < 0.5%. OR LCI and OR UCI are 95% confidence intervals of the odds ratios. ClinSigSimple data is provided by ClinVar where 0 = no current value of Likely pathogenic or Pathogenic and 1= at least one current record submitted with an interpretation of Likely pathogenic or Pathogenic (independent of whether that record includes assertion criteria and evidence). ^Upon visual inspection of the alignments, we find that the *UMOD* PTVs found in the WES data are part of a previously reported single complex inframe indel (<https://www.ncbi.nlm.nih.gov/clinvar/RCV000234799.1/>) that doesn’t result in haploinsufficiency. P-values were generated via two-tailed Fisher’s exact test. Included p-values are not corrected for multiple testing; the study-wide significance threshold is p≤2 ×10^-9^.

# Supplementary Table 4 – ExWAS Missense Variants, GWAS annotated

Provided as separate additional file

Study-wide significant (p ≤2x10 ^-9^) genotype-phenotype ExWAS associations (binary) for missense variants, showing alignment with variant associations from the EBI GWAS catalogue or ClinVar^1,11^ that are within 50kb on either side of each missense variant. P-values were generated via two-tailed Fisher’s exact test. Included p-values are not corrected for multiple testing; the study-wide significance threshold is p≤2 ×10^-9^.

# Supplementary Table 5 – ExWAS PTVs, GWAS annotated

Provided as separate additional file

Study-wide significant (p ≤2x10 ^-9^) genotype-phenotype associations (binary) from the exome-wide association study that are protein-truncating variants, showing alignment with variant associations from the EBI GWAS catalogue or ClinVar^1,11^ that are within 50kb on either side of each protein-truncating variant. P-values were generated via two-tailed Fisher’s exact test. Included p-values are not corrected for multiple testing; the study-wide significance threshold is p≤2 ×10^-9^.

# Supplementary Table 6 – Comparison between Fisher’s Exact Test, SAIGE and REGENIE

| **Measurable Metric** | **Fisher's Exact Test (FET)** | **SAIGE SPA 0.43** | **REGENIE 2.0.2 with Firth Approximation** |
| --- | --- | --- | --- |
| Median permutation-based genomic inflation factor [range] | 1.0006  [0.9675, 1.0698] | 0.9953 [0.9372,1.0940] | 1.0001 [0.9439,1.0602] |
| Number of tests with p-value < 2*10^^-9^ | 348 | 305 | 351 |
| Pearson's correlation with FET for all tests | NA | 0.9496 | 0.93911 |
| Pearson's correlation with SAIGE for all tests | 0.9496 | NA | 0.99202 |
| Pearson's correlation with FET for p-value < 1x10^^-8^ | NA | 0.9916 | 0.9884 |
| Pearson's correlation with SAIGE for p-value < 1x10^^-8^ | 0.9916 | NA | 0.9962 |
| Pearson's correlation with FET for p-value < 2*10^^-9^ | NA | 0.9925 | 0.9913 |
| Pearson's correlation with SAIGE for p-value < 2*10^^-9^ | 0.9925 | NA | 0.9965 |
| Number of tests performed* | 1,975M | 672M | 601M |
| CPU hours for all tests | 3,278.00 | 32,915.37 | 11,178.28 |
| CPU hours per test relative to FET | 1.00X | 29.51X | 11.21X |

**FET**: Fisher’s Exact test; **SPA**: Saddle-Point Approximation; **CPU**: Central Processing Unit. **NA**: Not Applicable. Accompanying figures in **Supplementary Figures 1 and 2.**
*For Fisher’s Exact all three genetic models were studied per variant-phenotype pair. For purposes of comparisons, the additive genetic model was adopted for SAIGE and REGENIE, explaining the reduced number of statistical tests performed. All autosomal variants were studied across 324 Chapter IX phenotypes

# Supplementary Table 7 – Lambda distributions from SAIGE, REGENIE, and Fisher’s exact test comparisons

Provided as separate additional file

For each trait in Chapter IX: median number of samples, median number of cases, median number of controls, control:case ratio, inflation factor of p-values from SAIGE, REGENIE and Fisher’s exact test, Pearson’s correlation coefficient between Phred scores from SAIGE, REGENIE and Fisher’s exact test once using all p-values and once using p-values < 0.01. Included p-values are not corrected for multiple testing; the study-wide significance threshold is p≤2 ×10^-9^.

# Supplementary Table 8 – Collapsing analysis top hits

Provided as separate additional file

Associations from gene-level rare variant collapsing analysis where p<1x10^-8^. There are 3,334 associations listed in total for binary traits of which 936 are significant and unique (i.e. counting associations that are significant in multiple models only once). There are 2,944 associations listed in total for quantitative traits of which 767 are significant and unique. P-values were generated via two-tailed Fisher’s exact test (binary traits) or linear regression (quantitative traits). Included p-values are not corrected for multiple testing; the study-wide significance threshold is p≤2 ×10^-9^.

# Supplementary Table 9 – Control-enriched collapsing analyses

| **model** | **phenotype** | **Gene** | **Case Freq** | **Ctrl Freq** | **p-value** | **OR** | **OR LCI** | **OR UCI** | **OMIM** |
| --- | --- | --- | --- | --- | --- | --- | --- | --- | --- |
| flexdmg | Source of report of K80 (cholelithiasis) | *ABCG5* | 0.22% | 0.66% | 2.6x10^-13^ | 0.336 | 0.238 | 0.475 | Sitosterolemia; 210250 (3); Autosomal recessive |
| ptv | 20002#1473#high cholesterol | *APOB* | 0.02% | 0.16% | 9.7 x10^-13^ | 0.151 | 0.075 | 0.305 | Hypercholesterolemia; due to ligand-defective apo B; 144010 (3); Autosomal dominant\|Hypobetalipoproteinemia; 615558 (3); Autosomal recessive |
| ptv5pcnt | 41202#L72#L72 Follicular cysts of skin and subcutaneous tissue | *FLG* | 9.27% | 12.06% | 1.2 x10^-11^ | 0.745 | 0.683 | 0.814 | Ichthyosis vulgaris; 146700 (3); Autosomal dominant\|{Dermatitis; atopic; susceptibility to; 2}; 605803 (3) |
| ptv5pcnt | 20002#1111#asthma | *IL33* | 0.80% | 1.18% | 4.8 x10^-10^ | 0.672 | 0.590 | 0.767 | . |
| ptv | 20002#1473#high cholesterol | *PCSK9* | 0.03% | 0.15% | 2.1 x10^-10^ | 0.201 | 0.107 | 0.378 | Hypercholesterolemia; familial; 3; 603776 (3)\|{Low density lipoprotein cholesterol level QTL 1}; 603776 (3) |
| flexdmg | Union#M10#M10 Gout | *SLC22A12* | 0.07% | 0.46% | 2.4 x10^-12^ | 0.148 | 0.070 | 0.312 | Hypouricemia; renal; 220150 (3); Autosomal recessive |

Summary of collapsing analysis results focusing on genes achieving a p<2x10^-9^ for control enrichment. Although for a given gene there might be multiple correlated phenotypes and correlated collapsing models that achieve significance here for a given gene, we present the phenotype and collapsing model combination that achieved the lowest odds ratio. The major histocompatibility complex (MHC) region was excluded. P-values were generated via two-tailed Fisher’s exact test. Included p-values are not corrected for multiple testing; the study-wide significance threshold is p≤2 ×10^-9^.

# Supplementary Table 10 – OMIM status of significant binary collapsing analysis associations

Provided as separate additional file

Genes with study-wide significant (p≤2x10^-9^) associations with binary traits in the collapsing analysis are annotated with their associated phenotype, where applicable, from OMIM^2^. Status: Known (phenotype in collapsing analysis is very similar to phenotype in OMIM); Expanded (phenotype in collapsing analysis is different to phenotype in OMIM); Novel (no phenotype in OMIM). P-values were generated via two-tailed Fisher’s exact test. Included p-values are not corrected for multiple testing; the study-wide significance threshold is p≤2 ×10^-9^.

# Supplementary Table 11 – Oncology aligned collapsing analyses summary

| **Gene** | **Category** | **Site(s)** |
| --- | --- | --- |
| *APC* | Solid | Digestive Organs |
| *ATM* | Solid | Digestive Organs |
| *BRCA1* | Solid | Breast, Female Genital Organs |
| *BRCA2* | Solid | Breast, Female Genital Organs, Male Genital Organs |
| *CDKN2A* | Solid | Skin |
| *CHEK2* | Solid | Breast |
| *FLG* | Solid | Skin |
| *MEN1* | Solid | Endocrine |
| *MLH1* | Solid | Digestive Organs |
| *MSH2* | Solid | Digestive Organs |
| *MSH6* | Solid | Digestive Organs, Female Genital Organs |
| *MUTYH* | Solid | Digestive Organs |
| *NF1* | Solid | Nervous System |
| *PALB2* | Solid | Breast |
| *PTPN14* | Solid | Skin |
| *RB1* | Solid | Eye |
| *ASXL1* | Haematological | Haematological |
| *DDX41* | Haematological | Haematological |
| *EZH2* | Haematological | Haematological |
| *IGLL5* | Haematological | Haematological |
| *SF3B1* | Haematological | Haematological |
| *SRSF2* | Haematological | Haematological |
| *TET2* | Haematological | Haematological |

Summary of collapsing analysis results focusing on genes achieving a p<2x10^-9^ in the Neoplasms Chapter. The full set of underlying oncology-aligned results can be found in **Supplementary Table 12**. Although for a given gene there might be multiple correlated phenotypes and correlated collapsing models that achieve significance here for a given gene, we summarise all the implicated sites. Secondary and benign neoplasms were excluded.

# Supplementary Table 12 – Oncology-related collapsing analysis associations

Provided as separate additional file

Study-wide significant (p<2x10^-9^) gene-phenotype associations with binary oncological traits in the collapsing analysis. These comprise both germline-driven hereditary solid tumours, and haematological malignancies even though the study was not designed to detect somatic variant-driven associations. P-values were generated via two-tailed Fisher’s exact test. Included p-values are not corrected for multiple testing; the study-wide significance threshold is p≤2 ×10^-^

# Supplementary Table 13A: Significant gene-phenotype relationships from collapsing analysis also identified in ExWAS

|  | **Total number of gene-phenotype relationships identified in the collapsing analysis** | **Number of gene-phenotype relationships from the collapsing analysis detected in ExWAS (%)** |
| --- | --- | --- |
| **Binary traits** | 724 | 125 (17.3%) |
| **Quantitative traits** | 767 | 446 (58.1%) |

The percentage of gene-phenotypes relationships (for both binary and quantitative traits) from the collapsing analysis that were also detected in the ExWAS. For binary traits, associations related to operation phenotypic codes (41200 and 41272) have been excluded for this comparison.

# Supplementary Table 13B: ExWAS associations (MAF<0.1%) also identified in collapsing analysis

When considering the rare PTV (MAF<0.1%) associations from ExWAS, we found that most of the gene-phenotype relationships were also captured in the PTV collapsing analysis (91% [83/91] for quantitative and 83% [30/36] for binary traits). The corresponding rates were lower for missense variants (45% [122/270] for quantitative and 23% [42/182] for binary traits) (**Supplementary Table 13B**).

|  | **Variant class** | **Number of gene-phenotype relationships detected in ExWAS (MAF<0.1%)** | **Number of gene-phenotype relationships from ExWAS (MAF<0.1%) detected in the collapsing analysis (%)** |
| --- | --- | --- | --- |
| **Binary traits** | **Missense variants plus PTVs** | 214 | 68 (31.8%) |
|  | **PTVs only** | 36 | 30 (83.3%) |
|  | **Missense variants only** | 182 | 42 (23.1%) |
| **Quantitative traits** | **Missense variants plus PTVs** | 345 | 182 (52.8%) |
|  | **PTVs only** | 91 | 83 (91.2%) |
|  | **Missense variants only** | 270 | 122 (45.2%) |

**Supplementary Table 13B: Significant gene-phenotype relationships from ExWAS (MAF<0.1%) that were identified in the collapsing analysis.** Summary of gene-phenotypes relationships (both binary & quantitative traits) identified in ExWAS (MAF<0.1%) that were also detected in the collapsing analysis. For binary traits, associations related to operation phenotypic codes (41200 and 41272) have been excluded for this comparison.

# Supplementary Table 14 – Non-intersecting PTV signals

Provided as separate additional file

Unique gene-trait pairs for statistically significant associations (p-value ≤ 2x10^-9^) discovered by two collapsing models that aggregate protein truncating variants with MAF < 0.1% and 5% ("ptv" and "ptv5pcnt", respectively). If the same gene-trait pair is found statistically significant (p-value ≤ 2x10^-9^) by single-variant tests (ExWAS) using a single protein truncating variant for any of our ExWAS models (genotypic, dominant, recessive), these associations are listed in column "ExWAS". If any collapsing or ExWAS associations do not reach our statistical significance thresholds, the equivalent cell is filled by "NA". P-values were generated via two-tailed Fisher’s exact test (binary traits) or linear regression (quantitative traits). Included p-values are not corrected for multiple testing; the study-wide significance threshold is p≤2 ×10^-9^. We observed that roughly 12% of the binary (49/410) and quantitative (83/670) trait associations detected in the PTV collapsing model that used a MAF filter of 5% were not captured in either the ExWAS or the rarer (MAF < 0.1%) PTV collapsing model.

# Supplementary Table 15 – Binary trait collapsing analyses in non-European populations

Provided as separate additional file

Associations from gene-level rare variant collapsing analysis in individuals of South Asian, East Asian or African ancestry, where p<1x10^-4^. P-values were generated via two-tailed Fisher’s exact test. Included p-values are not corrected for multiple testing; the study-wide significance threshold is p≤2 ×10^-9^.

# Supplementary Table 16 - Pan-ancestry binary collapsing analysis

Provided as separate additional file

Results of Cochran-Mantel-Haenszel (CMH) test to combine the results of the stratified binary trait collapsing analysis across the four studied ancestral groups, including the European population, where p<1x10^-4^. P-values for separate ancestries were generated via two-tailed Fisher’s exact test. P-values for pan-ancestry analysis were generated via CMH test. Included p-values are not corrected for multiple testing.

# Supplementary Table 17 - Pan-ancestry quantitative collapsing analysis

Provided as separate additional file

Results of regression-based pan-ancestry analysis incorporating four ancestral groups, including the European population, where p<1x10^-4^. P-values were generated by a linear regression model that included the following covariates: categorical ancestry (European, African, East Asian, or South Asian), the top five ancestry PC’s, age, and sex. Included p-values are not corrected for multiple testing.

# Supplementary Table 18 – ExWAS n-of-1 permutation summary

| *-of-1 Permutation p-value cut-off threshold* | *Binary ExWAS* | *Quantitative ExWAS* |
| --- | --- | --- |
| ***P < 1x10 ^-10^*** | *0* | *0* |
| ***P < 1x10 ^-9^*** | *4* | *2* |
| ***P < 1x10 ^-8^*** | *75* | *13* |
| *P < 1x10 ^-7^* | *684* | *155* |
| *P < 1x10 ^-6^* | *7582* | *1462* |
| *Total tests performed* | *~35.8 billion* | *~2.9 billion* |

The number of observations across the ~35.8 billion binary tests and ~2.9 billion quantitative tests (dominant model) at various p value thresholds.

# Supplementary Table 19 – ExWAS dominant model n-of-1 permutation results

Provided as separate additional file.

The 5000 lowest p-value signals from the n-of-1 ExWAS permutation analysis for both binary and quantitative traits. P-values were generated via two-tailed Fisher’s exact test (binary traits) or linear regression (quantitative traits). Included p-values are not corrected for multiple testing; the study-wide significance threshold is p≤2 ×10^-9^.

# Supplementary Table 20 – Collapsing analysis null distribution

Provided as separate additional file

Binary_Syn: The gene-phenotype associations (binary traits) from the synonymous negative control model of the collapsing analysis that have p<0.001. Quantitative_Syn: The gene-phenotype associations (quantitative traits) from the synonymous negative control model of the collapsing analysis that have p<0.001. Binary_perms: The 50 gene-phenotype associations (binary traits) from the n-of-1 permutation based collapsing analysis that have the lowest p values. Quantitative_perms: The 50 gene-phenotype associations (quantitative traits) from the n-of-1 permutation based collapsing analysis that have the lowest p values. P-values were generated via two-tailed Fisher’s exact test (binary traits) or linear regression (quantitative traits). Included p-values are not corrected for multiple testing; the study-wide significance threshold is p≤2 ×10^-9^.

# Supplementary Table 21 – Synonymous collapsing output

| *Synonymous collapsing output p-value cut-off threshold* | *Binary ExWAS* | *Quantitative ExWAS* |
| --- | --- | --- |
| ***P < 1x10 ^-10^*** | *1** | *1** |
| ***P < 1x10 ^-9^*** | *2** | *1** |
| ***P < 1x10 ^-8^*** | *2** | *2** |
| *P < 1x10 ^-7^* | *11* | *3* |
| *P < 1x10 ^-6^* | *105* | *30* |
| *P < 1x10 ^-5^* | *1,062* | *271* |
| *P < 1x10 ^-4^* | *10,967* | *2,417* |
| *P < 1x10 ^-3^* | *116,366* | *22,876* |
| *Total tests performed* | *~346.5 million* | *~28.3million* |

** Biological precedence (likely true) associations.*

Summary of the number of synonymous collapsing analysis associations at increasing p-value thresholds*.*

# Supplementary Table 22 – Lambda distributions

Provided as separate additional file

Lambda values for all gene-level collapsing analysis association tests.

# Supplementary Table 23 – Lambda ranges per collapsing model

The full set of lambdas for every studied phenotype-model combination are available in Supplementary Table 22 – lambda distributions. This table summarises the number and percentage of studied phenotypes (lambdas) falling between two specified lambda ranges, per model.

|  | **Binary traits** | | **Quantitative traits** | |
| --- | --- | --- | --- | --- |
| **Model** | **λ 0.95-1.05 (%)** | **λ 0.90-1.10 (%)** | **λ 0.95-1.05 (%)** | **λ 0.90-1.10 (%)** |
| **flexdmg** | 16,965 (93) | 18,273 (100) | 1,278 (90) | 1,342 (95) |
| **flexnonsyn** | 17,288 (94) | 18,272 (100) | 1,301 (92) | 1,357 (96) |
| **flexnonsynmtr** | 17,071 (93) | 18,273 (100) | 1,298 (91) | 1,367 (96) |
| **ptv** | 14,767 (81) | 17,966 (98) | 1,248 (88) | 1,338 (94) |
| **ptv5pcnt** | 15,098 (82) | 18,049 (99) | 1,238 (87) | 1,327 (94) |
| **ptvraredmg** | 16,752 (92) | 182,74 (100) | 1,283 (90) | 1,354 (95) |
| **raredmg** | 16,382 (90) | 18,252 (100) | 1,311 (92) | 1,388 (98) |
| **raredmgmtr** | 15,717 (86) | 18,149 (99) | 1,321 (93) | 1,402 (99) |
| **rec** | 12,490 (68) | 16,953 (93) | 1,216 (86) | 1,407 (99) |
| **syn** | 16,701 (91) | 18,265 (100) | 1,389 (98) | 1,416 (100) |
| **UR** | 15,316 (84) | 18,098 (99) | 1,273 (90) | 1,381 (97) |
| **URmtr** | 14,919 (82) | 17,928 (98) | 1,272 (90) | 1,386 (98) |

# Supplementary Table 24 – Gene Informativeness

Provided as separate additional file

Coverage statistics for each gene in CCDS release 22. Avg %10xCov Participant = percent of protein-coding sites covered with at least 10x coverage, averaged across all 269,171 UKB European participants.

# Supplementary Table 25 - Gene-level cautions

| Model | Phenotype | Gene | Case Qual | Case NoQual | Case Qual% | Ctrl Qual | Ctrl NoQual | Ctrl Qual% | Fisher’s Exact p-value |
| --- | --- | --- | --- | --- | --- | --- | --- | --- | --- |
| ptv5pcnt | Batch2 | *PRMT8* | 236 | 43857 | 0.54% | 7972 | 217106 | 3.54% | 0 |
| flexnonsyn | Batch2 | *OMA1* | 160 | 43933 | 0.36% | 0 | 225078 | 0.00% | 1.55E-126 |
| flexnonsynmtr | Batch2 | *OMA1* | 108 | 43985 | 0.24% | 0 | 225078 | 0.00% | 1.26E-85 |
| ptv5pcnt | Batch2 | *C1orf185* | 255 | 43838 | 0.58% | 3664 | 221414 | 1.63% | 4.77E-78 |
| ptv5pcnt | Batch2 | *C2orf76* | 19 | 44074 | 0.04% | 1298 | 223780 | 0.58% | 2.23E-74 |
| flexdmg | Batch2 | *OMA1* | 88 | 44005 | 0.20% | 0 | 225078 | 0.00% | 6.77E-70 |
| ptv5pcnt | Batch3 | *PRMT8* | 1929 | 42100 | 4.38% | 6279 | 218863 | 2.79% | 2.82E-64 |
| ptv5pcnt | Batch2 | *SKA3* | 955 | 43138 | 2.17% | 8208 | 216870 | 3.65% | 1.61E-61 |
| rec | Batch3 | *MUC5AC* | 110 | 43919 | 0.25% | 27 | 225115 | 0.01% | 7.12E-61 |
| ptvraredmg | Batch2 | *OMA1* | 73 | 44020 | 0.17% | 0 | 225078 | 0.00% | 4.22E-58 |
| ptvraredmg | Batch2 | *PRMT8* | 70 | 44023 | 0.16% | 1543 | 223535 | 0.69% | 1.47E-51 |
| flexdmg | Batch2 | *PRMT8* | 74 | 44019 | 0.17% | 1573 | 223505 | 0.70% | 3.98E-51 |
| flexnonsynmtr | Batch2 | *PRMT8* | 92 | 44001 | 0.21% | 1687 | 223391 | 0.75% | 4.67E-48 |
| flexnonsyn | Batch2 | *PRMT8* | 115 | 43978 | 0.26% | 1863 | 223215 | 0.83% | 1.74E-46 |
| ptv5pcnt | Batch2 | *ADAM2* | 328 | 43765 | 0.74% | 3278 | 221800 | 1.46% | 2.76E-37 |
| ptv | Batch2 | *PRMT8* | 30 | 44063 | 0.07% | 890 | 224188 | 0.40% | 5.55E-37 |
| syn | Batch2 | *OMA1* | 45 | 44048 | 0.10% | 0 | 225078 | 0.00% | 4.34E-36 |
| ptv5pcnt | Batch4 | *C2orf76* | 665 | 90623 | 0.73% | 635 | 171485 | 0.37% | 3.52E-34 |
| ptv5pcnt | Batch1 | *EP400* | 1513 | 88248 | 1.69% | 1932 | 172553 | 1.11% | 4.17E-34 |
| ptv5pcnt | Batch2 | *MUC6* | 487 | 43606 | 1.10% | 4240 | 220838 | 1.88% | 2.14E-33 |
| raredmg | Batch2 | *OMA1* | 37 | 44056 | 0.08% | 0 | 225078 | 0.00% | 8.42E-30 |
| ptv5pcnt | Batch2 | *OMA1* | 37 | 44056 | 0.08% | 0 | 225078 | 0.00% | 8.42E-30 |
| ptv | Batch2 | *OMA1* | 37 | 44056 | 0.08% | 0 | 225078 | 0.00% | 8.42E-30 |
| flexnonsyn | Batch4 | *OMA1* | 0 | 91288 | 0.00% | 157 | 171963 | 0.09% | 1.63E-29 |
| flexnonsyn | Batch1 | *OMA1* | 0 | 89761 | 0.00% | 157 | 174328 | 0.09% | 8.83E-29 |
| rec | Batch2 | *OMA1* | 33 | 44060 | 0.07% | 0 | 225078 | 0.00% | 1.17E-26 |
| ptv5pcnt | Batch2 | *ADAM29* | 284 | 43809 | 0.64% | 658 | 224420 | 0.29% | 9.95E-26 |
| ptv5pcnt | Batch4 | *PRMT8* | 3225 | 88063 | 3.53% | 4803 | 167317 | 2.79% | 1.76E-25 |
| ptv5pcnt | Batch2 | *CXCL11* | 44 | 44049 | 0.10% | 825 | 224253 | 0.37% | 1.07E-24 |
| ptv5pcnt | Batch1 | *ZC3H8* | 629 | 89132 | 0.70% | 699 | 173786 | 0.40% | 6.32E-24 |
| syn | Batch3 | *LPAR2* | 215 | 43814 | 0.49% | 453 | 224689 | 0.20% | 8.40E-24 |
| syn | Batch3 | *ROR1* | 217 | 43812 | 0.49% | 487 | 224655 | 0.22% | 2.02E-21 |
| ptv5pcnt | Batch2 | *OR6F1* | 147 | 43946 | 0.33% | 1558 | 223520 | 0.69% | 8.65E-21 |
| flexnonsynmtr | Batch4 | *OMA1* | 0 | 91288 | 0.00% | 107 | 172013 | 0.06% | 1.95E-20 |
| syn | Batch3 | *XPC* | 228 | 43801 | 0.52% | 538 | 224604 | 0.24% | 2.51E-20 |
| ptv5pcnt | Batch2 | *ZC3H8* | 107 | 43986 | 0.24% | 1239 | 223839 | 0.55% | 1.06E-19 |
| flexnonsynmtr | Batch1 | *OMA1* | 0 | 89761 | 0.00% | 106 | 174379 | 0.06% | 1.52E-19 |
| ptv5pcnt | Batch4 | *SKA3* | 3506 | 87782 | 3.84% | 5452 | 166668 | 3.17% | 2.56E-19 |
| flexnonsynmtr | Batch2 | *HIGD1B* | 68 | 44025 | 0.15% | 70 | 225008 | 0.03% | 3.87E-19 |
| raredmgmtr | Batch2 | *OMA1* | 23 | 44070 | 0.05% | 0 | 225078 | 0.00% | 8.47E-19 |
| ptv5pcnt | Batch4 | *EP400* | 938 | 90350 | 1.03% | 2461 | 169659 | 1.43% | 8.66E-19 |
| flexdmg | Batch4 | *OMA1* | 0 | 91288 | 0.00% | 87 | 172033 | 0.05% | 1.01E-16 |
| ptv5pcnt | Batch2 | *SREBF1* | 10 | 44083 | 0.02% | 348 | 224730 | 0.15% | 2.12E-16 |
| ptv5pcnt | Batch2 | *CADPS* | 56 | 44037 | 0.13% | 56 | 225022 | 0.02% | 2.13E-16 |
| flexdmg | Batch1 | *OMA1* | 0 | 89761 | 0.00% | 87 | 174398 | 0.05% | 2.46E-16 |
| ptv5pcnt | Batch4 | *CHIT1* | 366 | 90922 | 0.40% | 380 | 171740 | 0.22% | 6.78E-16 |
| syn | Batch3 | *METTL22* | 198 | 43831 | 0.45% | 502 | 224640 | 0.22% | 2.35E-15 |
| ptv5pcnt | Batch4 | *CIC* | 96 | 91192 | 0.11% | 46 | 172074 | 0.03% | 2.42E-15 |
| ptv5pcnt | Batch2 | *FASTKD1* | 2176 | 41917 | 4.94% | 13206 | 211872 | 5.87% | 4.33E-15 |
| ptv5pcnt | Batch3 | *ZC3H8* | 122 | 43907 | 0.28% | 1224 | 223918 | 0.54% | 8.30E-15 |
| ptv5pcnt | Batch1 | *HRCT1* | 2328 | 87433 | 2.59% | 3687 | 170798 | 2.11% | 8.55E-15 |
| ptv5pcnt | Batch4 | *FBRS* | 116 | 91172 | 0.13% | 69 | 172051 | 0.04% | 1.11E-14 |
| ptv5pcnt | Batch2 | *ZNF880* | 95 | 43998 | 0.22% | 171 | 224907 | 0.08% | 2.48E-14 |
| ptv5pcnt | Batch4 | *C1orf185* | 1560 | 89728 | 1.71% | 2293 | 169827 | 1.33% | 3.92E-14 |
| ptvraredmg | Batch4 | *OMA1* | 0 | 91288 | 0.00% | 72 | 172048 | 0.04% | 6.24E-14 |
| flexdmg | Batch4 | *HRCT1* | 2366 | 88922 | 2.59% | 5345 | 166775 | 3.11% | 6.50E-14 |
| ptvraredmg | Batch1 | *OMA1* | 0 | 89761 | 0.00% | 72 | 174413 | 0.04% | 1.39E-13 |
| ptvraredmg | Batch4 | *HRCT1* | 1902 | 89386 | 2.08% | 4367 | 167753 | 2.54% | 2.16E-13 |
| rec | Batch4 | *MUC5AC* | 9 | 91279 | 0.01% | 119 | 172001 | 0.07% | 2.30E-13 |
| ptv5pcnt | Batch2 | *AHCTF1* | 1153 | 42940 | 2.61% | 7342 | 217736 | 3.26% | 3.89E-13 |
| flexnonsynmtr | Batch4 | *HRCT1* | 2511 | 88777 | 2.75% | 5609 | 166511 | 3.26% | 4.88E-13 |
| flexnonsyn | Batch3 | *OMA1* | 0 | 44029 | 0.00% | 160 | 224982 | 0.07% | 6.34E-13 |
| ptv5pcnt | Batch4 | *WIPF3* | 132 | 91156 | 0.14% | 95 | 172025 | 0.06% | 6.38E-13 |
| flexnonsyn | Batch2 | *HIGD1B* | 101 | 43992 | 0.23% | 204 | 224874 | 0.09% | 9.46E-13 |
| ptv5pcnt | Batch3 | *SREBF1* | 113 | 43916 | 0.26% | 245 | 224897 | 0.11% | 1.49E-12 |
| syn | Batch3 | *FEM1B* | 217 | 43812 | 0.49% | 620 | 224522 | 0.28% | 2.09E-12 |
| ptv5pcnt | Batch2 | *HRCT1* | 807 | 43286 | 1.83% | 5314 | 219764 | 2.36% | 2.36E-12 |
| ptv5pcnt | Batch2 | *MAPKAPK2* | 4 | 44089 | 0.01% | 215 | 224863 | 0.10% | 2.89E-12 |
| UR | Batch2 | *OMA1* | 14 | 44079 | 0.03% | 0 | 225078 | 0.00% | 1.00E-11 |
| flexnonsyn | Batch4 | *HRCT1* | 2855 | 88433 | 3.13% | 6238 | 165882 | 3.62% | 2.29E-11 |
| ptv5pcnt | Batch4 | *HRCT1* | 1835 | 89453 | 2.01% | 4154 | 167966 | 2.41% | 2.65E-11 |
| flexdmg | Batch1 | *HRCT1* | 2909 | 86852 | 3.24% | 4844 | 169641 | 2.78% | 2.97E-11 |
| ptv5pcnt | Batch2 | *SCAF1* | 1 | 44092 | 0.00% | 156 | 224922 | 0.07% | 3.35E-11 |
| flexnonsynmtr | Batch1 | *HRCT1* | 3051 | 86710 | 3.40% | 5102 | 169383 | 2.92% | 3.35E-11 |
| syn | Batch3 | *SRC* | 174 | 43855 | 0.40% | 480 | 224662 | 0.21% | 3.52E-11 |
| ptv5pcnt | Batch2 | *TPTE2* | 218 | 43875 | 0.49% | 1735 | 223343 | 0.77% | 6.89E-11 |
| ptv5pcnt | Batch1 | *C1orf185* | 1496 | 88265 | 1.67% | 2351 | 172134 | 1.35% | 1.38E-10 |
| ptvraredmg | Batch1 | *HRCT1* | 2379 | 87382 | 2.65% | 3919 | 170566 | 2.25% | 1.56E-10 |
| ptv5pcnt | Batch4 | *GAGE10* | 273 | 91015 | 0.30% | 795 | 171325 | 0.46% | 1.58E-10 |
| ptv5pcnt | Batch2 | *CHIT1* | 64 | 44029 | 0.15% | 692 | 224386 | 0.31% | 2.34E-10 |
| ptv5pcnt | Batch4 | *CADPS* | 10 | 91278 | 0.01% | 100 | 172020 | 0.06% | 6.38E-10 |
| ptv5pcnt | Batch4 | *OR6F1* | 702 | 90586 | 0.77% | 973 | 171147 | 0.57% | 6.96E-10 |
| ptv5pcnt | Batch2 | *EP400* | 445 | 43648 | 1.01% | 3057 | 222021 | 1.36% | 1.24E-09 |
| rec | Batch1 | *MUC5AC* | 15 | 89746 | 0.02% | 120 | 174365 | 0.07% | 1.44E-09 |
| ptv5pcnt | Batch2 | *GOLGA6L2* | 102 | 43991 | 0.23% | 935 | 224143 | 0.42% | 1.58E-09 |
| ptv5pcnt | Batch1 | *NXNL1* | 739 | 89022 | 0.82% | 1076 | 173409 | 0.62% | 1.94E-09 |
| URmtr | Batch2 | *OMA1* | 11 | 44082 | 0.02% | 0 | 225078 | 0.00% | 2.28E-09 |
| flexnonsyn | Batch1 | *HRCT1* | 3369 | 86392 | 3.75% | 5769 | 168716 | 3.31% | 3.34E-09 |
| flexnonsyn | Batch2 | *NOSTRIN* | 232 | 43861 | 0.53% | 747 | 224331 | 0.33% | 3.48E-09 |
| ptv5pcnt | Batch2 | *DAZAP1* | 13 | 44080 | 0.03% | 262 | 224816 | 0.12% | 4.00E-09 |
| ptv5pcnt | Batch2 | *MTMR2* | 320 | 43773 | 0.73% | 2285 | 222793 | 1.02% | 4.60E-09 |
| ptv5pcnt | Batch1 | *FBRS* | 27 | 89734 | 0.03% | 156 | 174329 | 0.09% | 6.89E-09 |
| syn | Batch4 | *OMA1* | 0 | 91288 | 0.00% | 44 | 172076 | 0.03% | 8.76E-09 |
| flexnonsynmtr | Batch3 | *OMA1* | 0 | 44029 | 0.00% | 108 | 225034 | 0.05% | 9.35E-09 |
| syn | Batch1 | *OMA1* | 0 | 89761 | 0.00% | 45 | 174440 | 0.03% | 9.98E-09 |
| ptv5pcnt | Batch2 | *RECQL5* | 275 | 43818 | 0.62% | 937 | 224141 | 0.42% | 1.22E-08 |
| ptv5pcnt | Batch2 | *CHAC2* | 32 | 44061 | 0.07% | 413 | 224665 | 0.18% | 1.24E-08 |
| ptv5pcnt | Batch2 | *ATXN3* | 1015 | 43078 | 2.30% | 6243 | 218835 | 2.77% | 1.24E-08 |
| ptv5pcnt | Batch2 | *RNF44* | 38 | 44055 | 0.09% | 458 | 224620 | 0.20% | 1.27E-08 |
| ptv5pcnt | Batch2 | *NXNL1* | 216 | 43877 | 0.49% | 1634 | 223444 | 0.73% | 1.30E-08 |
| ptv5pcnt | Batch2 | *RBM44* | 183 | 43910 | 0.42% | 1427 | 223651 | 0.63% | 1.56E-08 |
| flexdmg | Batch3 | *MUC5AC* | 266 | 43763 | 0.60% | 905 | 224237 | 0.40% | 1.69E-08 |
| ptv5pcnt | Batch1 | *SKA3* | 3299 | 86462 | 3.68% | 5688 | 168797 | 3.26% | 2.99E-08 |
| ptv5pcnt | Batch4 | *ADAM2* | 1380 | 89908 | 1.51% | 2150 | 169970 | 1.25% | 3.33E-08 |
| ptv5pcnt | Batch4 | *MUC6* | 1783 | 89505 | 1.95% | 2847 | 169273 | 1.65% | 3.44E-08 |
| flexdmg | Batch4 | *PRMT8* | 666 | 90622 | 0.73% | 952 | 171168 | 0.55% | 5.57E-08 |
| ptv5pcnt | Batch3 | *GOLGA6L2* | 237 | 43792 | 0.54% | 800 | 224342 | 0.36% | 6.51E-08 |
| flexnonsynmtr | Batch2 | *NOSTRIN* | 123 | 43970 | 0.28% | 347 | 224731 | 0.15% | 6.60E-08 |
| ptv5pcnt | Batch2 | *CCDC34* | 59 | 44034 | 0.13% | 593 | 224485 | 0.26% | 7.41E-08 |
| rec | Batch2 | *PRB4* | 27 | 44066 | 0.06% | 355 | 224723 | 0.16% | 7.85E-08 |

Genes identified as being potentially susceptible to batch effects based on p<1x10^-7^ in collapsing analysis using sequencing batch as a phenotype. P-values were calculated using two-tailed Fisher’s exact test. Included p-values are not corrected for multiple testing; the study-wide significance threshold is p≤2 ×10^-9^.

# Supplementary Table 26 – Variant-level cautions

Provided as separate additional file

Variants identified as being potentially susceptible to batch effects based on p<1x10^-6^ in variant-level association tests using sequencing batch as a phenotype.

# Supplementary Table 27 - Variant-level exemptions

| Phenotype | Variant | Genetic model | Gene | Variant Effect | CaseGT | CtrlGT | p-value | ClinVar | Indication |
| --- | --- | --- | --- | --- | --- | --- | --- | --- | --- |
| Batch2 | 22-43928847-C-G | dominant | *PNPLA3* | missense | '27769/11380/1695' | '138616/73238/10486' | 1.9E-106 | drug response | Fatty liver disease, nonalcoholic 1 |
| Batch2 | 22-43928847-C-G | allelic | *PNPLA3* | missense | '27769/11380/1695' | '138616/73238/10486' | 1.6E-92 | drug response | Fatty liver disease, nonalcoholic 1 |
| Batch2 | 1-114693436-G-A | allelic | *AMPD1* | stop gained | '33872/7593/513' | '171950/46096/3494' | 7.2E-47 | Uncertain significance, other | Muscle AMP deaminase deficiency |
| Batch2 | 1-114693436-G-A | dominant | *AMPD1* | stop gained | '33872/7593/513' | '171950/46096/3494' | 2.6E-45 | Uncertain significance, other | Muscle AMP deaminase deficiency |
| Batch2 | 1-158618068-G-A | dominant | *SPTA1* | non coding transcript exon | '23376/14733/3098' | '117060/85315/17528' | 4.3E-39 | Conflicting interpretations of pathogenicity | Elliptocytosis 2; Hemolytic anemia; Hereditary pyropoikilocytosis; Spherocytosis type 3 |
| Batch2 | 1-158618068-G-A | allelic | *SPTA1* | non coding transcript exon | '23376/14733/3098' | '117060/85315/17528' | 5.8E-32 | Conflicting interpretations of pathogenicity | Elliptocytosis 2;Hemolytic anemia; Hereditary pyropoikilocytosis; Spherocytosis type 3 |
| Batch4 | 1-158618068-G-A | dominant | *SPTA1* | non coding transcript exon | '47145/35609/7214' | '90278/62355/12984' | 1.9E-24 | Conflicting interpretations of pathogenicity | Elliptocytosis 2;Hemolytic anemia; Hereditary pyropoikilocytosis; Spherocytosis type 3 |
| Batch4 | 22-43928847-C-G | dominant | *PNPLA3* | missense | '56054/30191/4225' | '106770/52661/7686' | 3.3E-22 | drug response | Fatty liver disease, nonalcoholic 1 |
| Batch4 | 1-158618068-G-A | allelic | *SPTA1* | non coding transcript exon | '47145/35609/7214' | '90278/62355/12984' | 1.7E-18 | Conflicting interpretations of pathogenicity | Elliptocytosis 2; Hemolytic anemia; Hereditary pyropoikilocytosis; Spherocytosis type 3 |
| Batch1 | 1-114693436-G-A | allelic | *AMPD1* | stop gained | '68400/18830/1458' | '133614/33937/2483' | 4.0E-18 | Uncertain significance, other | Muscle AMP deaminase deficiency |
| Batch4 | 1-114693436-G-A | dominant | *AMPD1* | stop gained | '69607/19170/1448' | '131803/33405/2477' | 2.1E-17 | Uncertain significance, other | Muscle AMP deaminase deficiency |
| Batch1 | 1-114693436-G-A | dominant | *AMPD1* | stop gained | '68400/18830/1458' | '133614/33937/2483' | 2.3E-17 | Uncertain significance, other | Muscle AMP deaminase deficiency |
| Batch4 | 22-43928847-C-G | allelic | *PNPLA3* | missense | '56054/30191/4225' | '106770/52661/7686' | 3.0E-17 | drug response | Fatty liver disease, nonalcoholic 1 |
| Batch4 | 1-114693436-G-A | allelic | *AMPD1* | stop gained | '69607/19170/1448' | '131803/33405/2477' | 4.7E-17 | Uncertain significance, other | Muscle AMP deaminase deficiency |
| Batch3 | 1-114693436-G-A | dominant | *AMPD1* | stop gained | '33943/8096/588' | '171879/45593/3419' | 6.6E-17 | Uncertain significance, other | Muscle AMP deaminase deficiency |
| Batch3 | 1-114693436-G-A | allelic | *AMPD1* | stop gained | '33943/8096/588' | '171879/45593/3419' | 9.2E-17 | Uncertain significance, other | Muscle AMP deaminase deficiency |
| Batch2 | 16-16208729-T-C | allelic | *ABCC6* | missense | '43166/0/2' | '215119/93/85' | 3.5E-16 | Conflicting interpretations of pathogenicity | Pseudoxanthoma elasticum |
| Batch2 | 1-158627717-G-C | dominant | *SPTA1* | missense | '22960/15230/3547' | '116768/85870/17813' | 1.7E-14 | Benign/Likely benign | Elliptocytosis 2;Hemolytic anemia; Hereditary pyropoikilocytosis; Spherocytosis type 3 |
| Batch2 | X-106034370-C-A | recessive | *SERPINA7* | missense | '18519/4740/319/ 16366/1775' | '94850/25264/1780/ 88231/11402' | 3.4E-14 | Pathogenic | Thyroxine-binding globulin, variant P |
| Batch2 | 16-16208729-T-C | dominant | *ABCC6* | missense | '43166/0/2' | '215119/93/85' | 7.0E-12 | Conflicting interpretations of pathogenicity | Pseudoxanthoma elasticum |
| Batch3 | 7-87600185-T-C | recessive | *ABCB1* | 5 prime UTR | '486/6695/36620' | '1747/34879/187339' | 2.4E-11 | drug response | Tramadol response |
| Batch2 | 12-10118488-A-C | dominant | *CLEC7A* | stop gained | '36765/5846/271' | '188806/32886/1531' | 9.0E-10 | Benign | Aspergillosis, susceptibility to;Familial chronic mucocutaneous candidiasis |
| Batch2 | 5-13916424-A-G | allelic | *DNAH5* | missense | '30303/47/0' | '216621/753/4' | 1.4E-09 | Uncertain significance | Ciliary dyskinesia, primary, 3;Primary ciliary dyskinesia |
| Batch2 | 5-13916424-A-G | dominant | *DNAH5* | missense | '30303/47/0' | '216621/753/4' | 1.7E-09 | Uncertain significance | Ciliary dyskinesia, primary, 3;Primary ciliary dyskinesia |
| Batch2 | 12-10118488-A-C | allelic | *CLEC7A* | stop gained | '36765/5846/271' | '188806/32886/1531' | 1.7E-09 | Benign | Aspergillosis, susceptibility to;Familial chronic mucocutaneous candidiasis |
| Batch3 | 16-16208729-T-C | allelic | *ABCC6* | missense | '41621/32/24' | '216664/61/63' | 1.5E-08 | Conflicting interpretations of pathogenicity | Pseudoxanthoma elasticum |
| Batch4 | 12-21178615-T-C | allelic | *SLCO1B1* | missense | '65439/22940/2110' | '123715/41250/3712' | 2.0E-08 | drug response | Gilbert's syndrome;Rotor syndrome |
| Batch4 | 12-21178615-T-C | dominant | *SLCO1B1* | missense | '65439/22940/2110' | '123715/41250/3712' | 2.1E-08 | drug response | Gilbert's syndrome;Rotor syndrome |
| Batch2 | 1-114693436-G-A | recessive | *AMPD1* | stop gained | '33872/7593/513' | '171950/46096/3494' | 2.5E-08 | Uncertain significance, other | Muscle AMP deaminase deficiency |
| Batch3 | 4-112623837-G-T | allelic | *ZGRF1* | missense | '41541/441/2' | '216937/3018/19' | 3.7E-08 | Likely pathogenic | Speech-language disorder 1 |
| Batch3 | 4-112623837-G-T | dominant | *ZGRF1* | missense | '41541/441/2' | '216937/3018/19' | 4.5E-08 | Likely pathogenic | Speech-language disorder 1 |
| Batch3 | 1-231272345-A-G | allelic | *GNPAT* | missense | '26218/13429/1943' | '134324/72497/10865' | 9.9E-08 | Benign | Rhizomelic chondrodysplasia punctata type 2;not provided |
| Batch3 | 1-231272345-A-G | dominant | *GNPAT* | missense | '26218/13429/1943' | '134324/72497/10865' | 2.8E-07 | Benign | Rhizomelic chondrodysplasia punctata type 2;not provided |
| Batch2 | 22-43928847-C-G | recessive | *PNPLA3* | missense | '27769/11380/1695' | '138616/73238/10486' | 4.2E-07 | drug response | Fatty liver disease, nonalcoholic 1 |
| Batch4 | 1-158627717-G-C | dominant | *SPTA1* | missense | '47130/35247/7211' | '89612/63733/13682' | 4.4E-07 | Benign/Likely benign | Elliptocytosis 2; Hemolytic anemia; Hereditary pyropoikilocytosis; Spherocytosis type 3 |
| Batch3 | 16-16208729-T-C | dominant | *ABCC6* | missense | '41621/32/24' | '216664/61/63' | 6.8E-07 | Conflicting interpretations of pathogenicity | Pseudoxanthoma elasticum |
| Batch2 | 4-112623837-G-T | allelic | *ZGRF1* | missense | '42240/461/2' | '216238/2998/19' | 7.0E-07 | Likely pathogenic | Speech-language disorder 1 |
| Batch2 | 2-218882368-C-A | dominant | *WNT10A* | stop gained | '43843/249/0' | '224192/881/0' | 8.3E-07 | Pathogenic | Inborn genetic diseases;Odonto-onycho-dermal dysplasia;WNT10A-Related Disorders |
| Batch2 | 4-112623837-G-T | dominant | *ZGRF1* | missense | '42240/461/2' | '216238/2998/19' | 8.5E-07 | Likely pathogenic | Speech-language disorder 1 |
| Batch2 | 2-218882368-C-A | allelic | *WNT10A* | stop gained | '43843/249/0' | '224192/881/0' | 8.5E-07 | Pathogenic | Inborn genetic diseases;Odonto-onycho-dermal dysplasia;WNT10A-Related Disorders |
| Batch4 | 4-112623837-G-T | allelic | *ZGRF1* | missense | '88611/1321/12' | '164370/2078/9' | 9.8E-07 | Likely pathogenic | Speech-language disorder 1 |

Thirteen variants identified as being potentially susceptible to batch effects, but were retained in ExWAS analyses on basis of overlapping with ClinVar curated variants. P-values were generated via two-tailed Fisher’s exact test. Included p-values are not corrected for multiple testing; the study-wide significance threshold is p≤2 ×10^-9^.

# Supplementary Table 28 – Cross-study comparison of 50K UKB exomes

Provided as separate additional file

Unique gene-trait pairs for associations discovered by Regeneron, Helix and AstraZeneca using the first tranche of 50,000 exomes from UK Biobank. We only report associations with p-value<3.4x10^-10^, which is the significance threshold adopted by Helix. For the results from Helix and AstraZeneca, if the same gene-trait pair is found significant for different models (e.g. ptv versus coding for Helix), we used the pair with the highest statistical significance. For determining which traits were analysed by Regeneron, we used the 215 quantitative & 1023 binary traits listed in their Supplementary Table TraitsLists.xlsx.^3^ For Helix, we used the significant associations from the UK Biobank using European samples, found in Supplementary Data 2 from their manuscript, whereas all traits run by Helix are listed in their Supplementary Data 1.^4^ P-values were generated via two-tailed Fisher’s exact test (binary traits) or linear regression (quantitative traits). Included p-values are not corrected for multiple testing.

# Supplementary Table 29 - Correlated Chapter IX phenotypes

Provided as separate additional file

Phenotype pairs in Chapter IX for which Pearson’s *r*^2^ > 0.2.

# Supplementary References

1 Landrum, M. J. *et al.* ClinVar: improving access to variant interpretations and supporting evidence. *Nucleic Acids Res* **46**, D1062-D1067, doi:10.1093/nar/gkx1153 (2018).

2 Amberger, J. S., Bocchini, C. A., Schiettecatte, F., Scott, A. F. & Hamosh, A. OMIM.org: Online Mendelian Inheritance in Man (OMIM(R)), an online catalog of human genes and genetic disorders. *Nucleic Acids Res* **43**, D789-798, doi:10.1093/nar/gku1205 (2015).

3 Van Hout, C. V. *et al.* Exome sequencing and characterization of 49,960 individuals in the UK Biobank. *Nature* **586**, 749-756, doi:10.1038/s41586-020-2853-0 (2020).

4 Cirulli, E. T. *et al.* Genome-wide rare variant analysis for thousands of phenotypes in over 70,000 exomes from two cohorts. *Nat Commun* **11**, 542, doi:10.1038/s41467-020-14288-y (2020).

5 Loh, P. R., Kichaev, G., Gazal, S., Schoech, A. P. & Price, A. L. Mixed-model association for biobank-scale datasets. *Nat Genet* **50**, 906-908, doi:10.1038/s41588-018-0144-6 (2018).

6 Zhou, W. *et al.* Efficiently controlling for case-control imbalance and sample relatedness in large-scale genetic association studies. *Nat Genet* **50**, 1335-1341, doi:10.1038/s41588-018-0184-y (2018).

7 Millard, L. A. C., Davies, N. M., Gaunt, T. R., Davey Smith, G. & Tilling, K. Software Application Profile: PHESANT: a tool for performing automated phenome scans in UK Biobank. *Int J Epidemiol* **47**, 29-35, doi:10.1093/ije/dyx204 (2018).

8 Bycroft, C. *et al.* The UK Biobank resource with deep phenotyping and genomic data. *Nature* **562**, 203-209, doi:10.1038/s41586-018-0579-z (2018).

9 Mbatchou, J. *et al.* Computationally efficient whole-genome regression for quantitative and binary traits. *Nat Genet* **53**, 1097-1103, doi:10.1038/s41588-021-00870-7 (2021).

10 Traynelis, J. *et al.* Optimizing genomic medicine in epilepsy through a gene-customized approach to missense variant interpretation. *Genome Res* **27**, 1715-1729, doi:10.1101/gr.226589.117 (2017).

11 Buniello, A. *et al.* The NHGRI-EBI GWAS Catalog of published genome-wide association studies, targeted arrays and summary statistics 2019. *Nucleic Acids Res* **47**, D1005-D1012, doi:10.1093/nar/gky1120 (2019).
