# Supplementary material for: Rare variant contribution to human disease in 281,104 UK Biobank exomes
Source: Nature. 2021 Aug 10;597(7877):527–32. doi: 10.1038/s41586-021-03855-y (PMC8458098; doi:10.1038/s41586-021-03855-y)
Supplement: Supplementary file 3 — This file contains Supplementary Tables 1, 2, 4, 5, 7, 8, 10, 12, 14, 15-17, 19, 20, 22, 24, 26, 28 and 29. [file 41586_2021_3855_MOESM3_ESM.zip › Supplementary Table Guide.pdf]

Supplementary Datasets
Supplementary Table 1: Details of all phenotypes studied in this dataset.
Supplementary Table 2: Most significant genotype-phenotype associations from variant-level exome-wide association study.
Supplementary Table 4: Significant missense variants from the binary trait ExWAS annotated with known GWAS associations within 50 kilobases.
Supplementary Table 5: Significant PTVs from the binary trait ExWAS annotated with known GWAS associations within 50 kilobases.
Supplementary Table 7: Lambda distributions from SAIGE, REGENIE, and Fisher's exact test comparisons
Supplementary Table 8: Collapsing analysis top hits
Supplementary Table 10: OMIM status of significant binary collapsing analysis hassociations
Supplementary Table 12: Oncology-related collapsing analysis associations
Supplementary Table 14: Non-intersecting PTV signals between ExWAS and collapsing analyses
Supplementary Table 15: Binary trait collapsing analyses in non-European populations
Supplementary Table 16: Pan-ancestry binary collapsing analysis
Supplementary Table 17: Pan-ancestry quantitative collapsing analysis
Supplementary Table 19: ExWAS dominant model n-of-1 permutation results
Supplementary Table 20: Collapsing analysis null distribution
Supplementary Table 22: Lambda distributions from collapsing analyses
Supplementary Table 24: Gene informativeness
Supplementary Table 26: Variant-level cautions
Supplementary Table 28: Cross-study comparison of 50K UKB exomes
Supplementary Table 29: Correlated Chapter XI phenotypes
